# *C. elegans* Germ Cells Show Temperature and Age-Dependent Expression of *Cer1*, a Gypsy/Ty3-Related Retrotransposon

**DOI:** 10.1371/journal.ppat.1002591

**Published:** 2012-03-29

**Authors:** Shannon Dennis, Ujwal Sheth, Jessica L. Feldman, Kathryn A. English, James R. Priess

**Affiliations:** 1 Fred Hutchinson Cancer Research Center, Seattle, Washington, United States of America; 2 Howard Hughes Medical Institute, Seattle, Washington, United States of America; 3 Molecular and Cellular Biology Program, University of Washington, Seattle, Washington, United States of America; University of California Riverside, United States of America

## Abstract

Virus-like particles (VLPs) have not been observed in *Caenorhabditis* germ cells, although nematode genomes contain low numbers of retrotransposon and retroviral sequences. We used electron microscopy to search for VLPs in various wild strains of *Caenorhabditis*, and observed very rare candidate VLPs in some strains, including the standard laboratory strain of *C. elegans*, N2. We identified the N2 VLPs as capsids produced by *Cer1*, a retrotransposon in the Gypsy/Ty3 family of retroviruses/retrotransposons. *Cer1* expression is age and temperature dependent, with abundant expression at 15°C and no detectable expression at 25°C, explaining how VLPs escaped detection in previous studies. Similar age and temperature-dependent expression of *Cer1* retrotransposons was observed for several other wild strains, indicating that these properties are common, if not integral, features of this retroelement. Retrotransposons, in contrast to DNA transposons, have a cytoplasmic stage in replication, and those that infect non-dividing cells must pass their genomic material through nuclear pores. In most *C. elegans* germ cells, nuclear pores are largely covered by germline-specific organelles called P granules. Our results suggest that *Cer1* capsids target meiotic germ cells exiting pachytene, when free nuclear pores are added to the nuclear envelope and existing P granules begin to be removed. In pachytene germ cells, *Cer1* capsids concentrate away from nuclei on a subset of microtubules that are exceptionally resistant to microtubule inhibitors; the capsids can aggregate these stable microtubules in older adults, which exhibit a temperature-dependent decrease in egg viability. When germ cells exit pachytene, the stable microtubules disappear and capsids redistribute close to nuclei that have P granule-free nuclear pores. This redistribution is microtubule dependent, suggesting that capsids that are released from stable microtubules transfer onto new, dynamic microtubules to track toward nuclei. These studies introduce *C. elegans* as a model to study the interplay between retroelements and germ cell biology.

## Author Summary

Retrotransposons and retroviruses pose enormous threats to animal and plants because of their ability to insert into host genes. Retroelements that replicate in germ cells can, if left unchecked, expand exponentially in the host genome. *C. elegans* has proven to be an exceptional model system for studying many facets of cell and molecular biology, and the genome contains both retrotransposon and retroviral sequences. However, no virus-like particles have been observed in *C. elegans* germ cells. We show here that *Cer1*, an endogenous Gypsy/Ty3 class retrotransposon, is expressed at very high levels in *C. elegans* germ cells, but escaped detection in previous studies because its expression is both temperature and age dependent. These studies reveal new aspects of microtubule regulation in *C. elegans* that the retroelement appears to exploit to navigate the germ cell cytoplasm, and demonstrate the power of *C. elegans* for studying host/pathogen interactions in germ cell biology.

## Introduction

DNA transposons, retrotransposons, and retroviruses that are expressed in germ cells have tremendous potential to damage the genome by creating novel insertions that are transmitted vertically to host progeny. Because DNA transposons replicate by an excision and reintegration mechanism (“cut and paste”), replication of an endogenous element does not necessarily increase copy number [1]. Retrotransposons, however, replicate by first transcribing genomic RNAs that are later reverse transcribed for integration (“copy-and paste”), and endogenous elements thus have the potential to increase their copy numbers exponentially if left unchecked [1], [2]. Accordingly, animal and plant genomes typically contain far more copies of retrotransposons than of DNA transposons. For example, retrotransposons constitute about 40% and 75% of the human and *Zea mays* genomes, respectively, while DNA transposons contribute 3% and 8.6% [3], [4]. In striking contrast, retrotransposons constitute only 0.6% of the *C. elegans* genome, while DNA transposons make up about 12% 5–7. Although the *C. elegans* genome contains about 20 distinct families of long terminal repeat (LTR) retrotransposons and retroviruses, each family consists of only one or a few members [8]. Thus, retroelements enter the nematode genome at some frequency, but show comparatively little expansion. No viruses or Virus-Like Particles (VLPs) have ever been reported in the germ cells of *C. elegans* or other *Caenorhabditis* species, and experiments designed to identify spontaneous germline mutations in *C. elegans* yielded only novel insertions by DNA transposons [9], [10]. Indeed, *C. elegans* has long been considered an inadequate model organism to study natural virus-host interactions, although recent studies have shown that it is possible to artificially infect nematodes or nematode cell lines with promiscuous viruses, and a natural virus that infects intestinal cells has been identified [11]–[15].

A fundamental difference between DNA transposons and retroelements is that DNA transposons undergo their replication cycle entirely within the nucleus, while retrotransposons and retroviruses that infect non-dividing cells must transport their genetic material across nuclear pores. Non-LTR retrotransposons replicate by first producing a genomic RNA that is exported to the cytoplasm. This mRNA forms a ribonucleoprotein particle (RNP) that is imported back to the nucleus for reverse transcription and integration [16]. LTR retrotransposons closely resemble retroviruses, and encode similar GAG proteins (capsid, nucleocapsid) and POL proteins (protease, reverse transcriptase, ribonuclease H, integrase). Like retroviruses, LTR retrotransposons synthesize and export a genomic RNA that combines with capsid and nucleocapsid proteins to form VLPs. Some retroviruses require nuclear envelope breakdown to target host chromosomes, while others such as human immunodeficiency virus (HIV) infect non-dividing cells via entry through nuclear pores [17]. For example, nuclear entry of the HIV pre-integration complex involves the host nucleoporins NUP153 and RANBP2, and the karyopherin Transportin-3 [18], [19]. The HIV Vpr protein can interact directly with FG-repeat (phenylalanine glycine) nucleoporins, and these interactions appear necessary for the preintegration complex to be imported across nuclear pores and into the nucleus.

In *Caenorhabditis* germ cells, nuclear pores are intimately connected with large, germline-specific granules called P granules [20]. Many animals from nematodes to mammals contain distinctive granules in their germ cells that are not found in somatic cells; these granules can be either cytoplasmic or associated with nuclei, and their presence, localization, and morphology can change during germ cell development [21], [22]. However, in contrast to most examples of germ granules in other animals, P granules are found in *Caenorhabditis* germ cells throughout the life cycle, and are nearly always perinuclear [21], [22]. Moreover P granules cover at least 75% of the nuclear pores on most germ nuclei in the gonad [20]. Like nuclear pores, P granules contain multiple FG-repeat proteins, and can block the passive diffusion of molecules above 40 kDa [23], [24]. Thus, most molecules entering nematode germ nuclei must first traffic through 200–400 nm P granules before they reach the 50 nm nuclear pores. Specific import and export pathways involving FG-repeat nucleoporins are required to move most materials through nuclear pores, and we have proposed that related pathways involving FG-repeat P granule proteins might be necessary to traffic materials through the much larger P granules [24], [25].

*C. elegans* P granules consist of diverse molecules involved in mRNA metabolism [22], and have been shown to transiently intercept nascent mRNA in adult germ cells [24]. Thus, P granule localization over nuclear pores likely is important for regulatory proteins and RNAs within P granules to survey nascent mRNA before that mRNA is released into the large, syncytial cytoplasm of the gonad. However, we are interested in the possibility that P granule localization might secondarily restrict retroelements that, unlike DNA transposons, must access nuclear pores to complete their replication cycle. If so, nematode retroelements might require strategies to penetrate or bypass P granules. To begin a study of the cell biology of retroelements in nematode germ cells, we first searched for wild strains with VLPs in germ cells. We found VLPs in the germ cells of *C. japonica*, where they appear to form, or accumulate, directly on P granules. We also discovered that several strains of *C. elegans* have VLPs, including the standard laboratory strain N2, but only when adults are cultured at temperatures below 25°C. We show that the *C. elegans* VLPs are products of an endogenous, Gypsy/Ty3 class retrotransposon called *Cer1* (*C. elegans* retrotransposon 1) *Cer1* capsids are first detected near meiotic germ cells in early pachytene, but accumulate around nuclei only in late pachytene/diplotene when new nuclear pores are added that are not associated with P granules. We propose that *Cer1* capsids target this specific population of germ nuclei by exploiting changes in the stability of germ cell microtubules.

## Results

### Background

**Figure 1:**
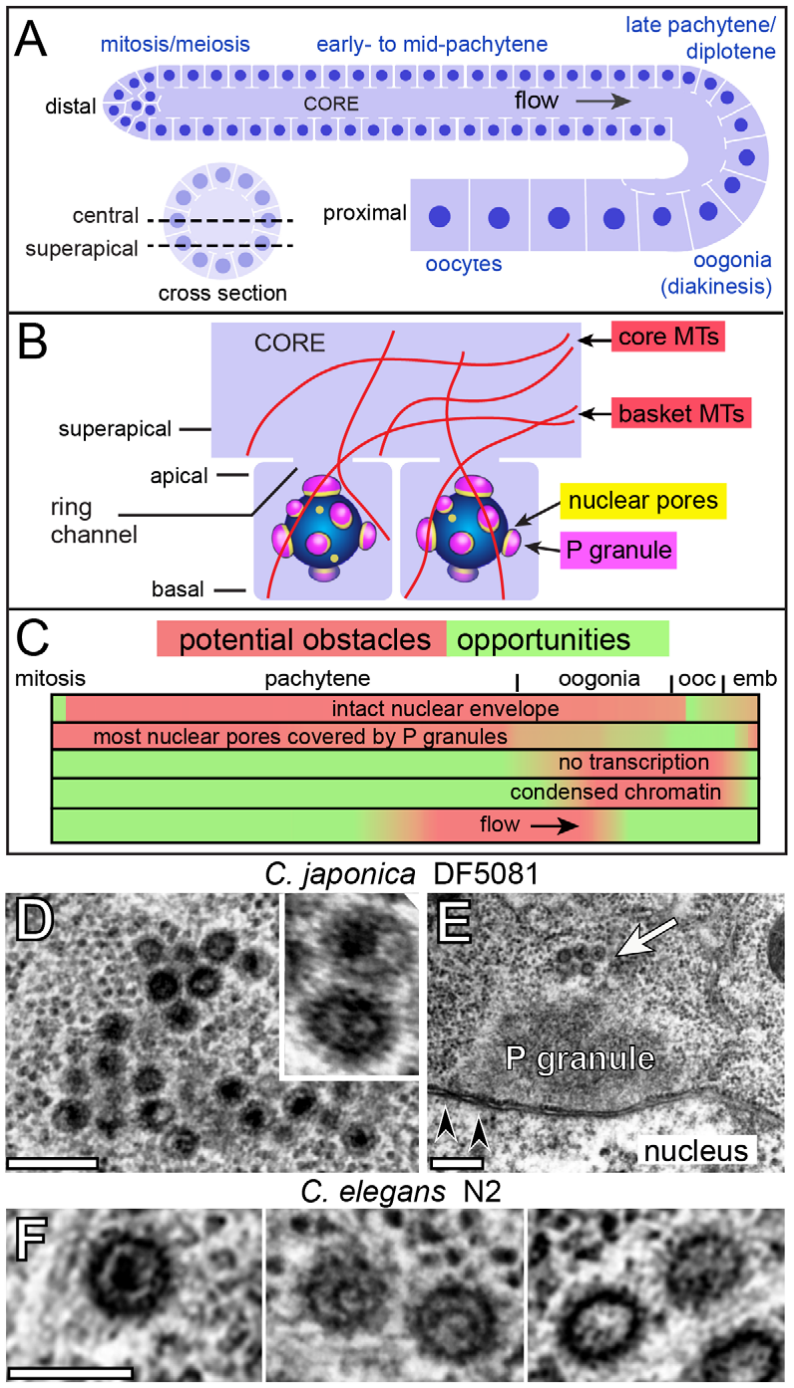
Nematode gonads contain VLPs. (A) Diagrams of longitudinal and cross sections through one arm of the adult hermaphrodite gonad with germ nuclei indicated in dark blue; somatic sheath cells that surround the gonad are not shown. Optical sectioning planes referred to in this paper are indicated in the cross-section (dashed lines). During development, germ cells move from the distal (mitotic) end of the gonad toward the proximal end, where they differentiate as oocytes. (B) Enlarged diagram of two germ cells with ring channels opening to the gonad core. Basket and core microtubules (MTs) are drawn in red. (C) Linear representation of germ cell development. Red shading indicates region-specific conditions in germ cells that might impede viral replication. These include the state of the nuclear envelope [67], P granules [21], transcription [68], chromatin [69], and cytoplasmic flow [27]; ooc = oocytes, emb = embryos. (D–E) Electron micrographs of *C. japonica* VLPs. Low magnification in panel E shows a small cluster of VLPs (arrow) on top of a P granule; arrowheads indicate examples of nuclear pores. See also Supplemental Figure S1A, S1B, S1C). (F) *C. elegans* VLPs showing variation in internal electron density; note curved, rod-like bodies within the VLPs in the middle panel. Scale bars: D (0.2 µm), E (0.5 µm), F (0.1 µm).

Each arm of the *C. elegans* adult hermaphrodite gonad resembles a U-shaped cylinder, with approximately 1000 germ nuclei near the periphery (Figure 1A) [26]. Most germ nuclei are incompletely cellularized by plasma membranes; the apical plasma membrane has a small opening, called the ring channel, that connects with a large, shared region of gonad cytoplasm called the core (Figure 1B). All images shown in this study are either longitudinal, optical sections taken through the center of the core (central plane) or taken slightly interior to the apical surface (superapical plane; Figure 1A, B). The basal plasma membrane contacts either extracellular matrix, or somatic sheath cells that partially surround the gonad. Although most of the gonad is a syncytium, by convention a nucleus and the surrounding cytoplasm extending up to the ring channel is referred to as a germ “cell”. Cells at the distal end of the gonad divide mitotically, and their descendants enter successive stages of meiosis and oogenesis as they progress toward the opposite, or proximal, end of the gonad. Actin-dependent, cytoplasmic flow transports materials along the gonad core, toward and into enlarging oogonia [27]. When oogonia reach their full size, their ring channels close to form cellularized oocytes. Dense populations of microtubules are present throughout the gonad, both within germ cells and in the gonad core. Pachytene nuclei are surrounded by a basket of microtubules (“basket microtubules”) that extend from the basal and lateral membranes, through the ring channel, and into the gonad core (Figure 1B) [27], [28]. By electron microscopy, other microtubules we call core microtubules appear to extend from the core plasma membrane into the superapical zone, or into deeper regions of the core, without entering germ cells (data not shown). Additional anatomical and physiological features of the gonad that are noteworthy as potential obstacles, or opportunities, for retroelements targeting germ cell chromosomes are summarized in Figure 1C.

### Germline VLPs in the N2 laboratory strain are the product of the *Cer1* retrotransposon

Electron microscopy was used to screen for candidate VLPs in the adult gonads of 21 *Caenorhabditis* strains. These included both *elegans* and non-*elegans* species, and both hermaphroditic and male/female strains (Table 1, and Materials and Methods). Representative germ cells were analyzed from mitosis through all stages of meiosis, up to the formation of cellularized oocytes; over 17,000 germ cells were scored in the initial survey, and all candidate VLPs were photographed. In these studies, we observed multiple examples of two types of atypical, spherical bodies we considered possible VLPs; two additional nuclear and cytoplasmic bodies were observed in some strains, but these occurred in less than 0.1% of germ cells and were not analyzed further.

**Table 1:** Electron microscopy of VLPs in *Caenorhabditis*.

Strain (20–23°C)	Locality	VLP-positive germ cells (%)	Germ cells scored
*C.e* N2 (lab stock) “Wild type”	Bristol, England	3.9	563
*C.e* N2 Reference	Bristol, England	6.0	941
*C.e* N2 Reference males	Bristol, England	0.0	955
*C.e* N2 (ancestral)	Bristol, England	3.9	933
*C.e* CB4853	Altadena, CA	0.0	586
*C.e* CB4854	Altadena, CA	0.0	774
*C.e* CB4555	Pasadena, CA	0.0	681
*C.e* CB4858	Pasadena, CA	0.0	915
*C.e* DH424	El Prieto Canyon, CA	0.0	808
*C.e* CB4856	Hawaii	0.0	1172
*C.e* KR314	Vancouver, Canada	4.6	326
*C.e* JU322	Merlet, France	3.7	406
*C.e* JU263	Le Blanc, Indre, France	0.0	871
*C.e* RW7000	Bergerac, France	0.0	745
*C.e* CB4851	Bergerac, France	0.0	620
*C.e* AB1	Adelaide, Australia	0.0	629
*C.e* JU258	Ribeiro Frio, Madeira	0.0	745
*C.e* MY2	Mecklenbeck, Germany	0.9	544
*C.e* RC301	Frieburg, Germany	0.0	889
*C. brenneri* PB2801	Costa Rica	0.0	610
*C. briggsae* AF16	Ahmedabad, India	0.0	657
*C. japonica* DF5081	Takeo, Japan	19.3	530
*C. japonica* DF5081 males	Takeo, Japan	16.2	378
*C. remanei* EM464	Brooklyn, NY	0.0	630
*C. remanei* SB146	Freiburg, Germany	0.0	561

The first type of VLP was observed only in *C. japonica*. These VLPs are about 80 nm in diameter, covered by fine spikes, and have variable, highly electron-dense cores (Figure 1D). The *C. japonica* VLPs were found in all female and male gonads examined (n>30 for each), and were present in 16–19% of total germ cells beginning in the mid-pachytene region (Table 1). From this region through early oogenesis, essentially all VLPs were adjacent to P granules (Figure 1E and Supplemental Figure S1A, S1B, S1C). The VLPs were present in grape-like clusters that generally increased in size with germ cell development. In oogonia, where P granules detach from the nucleus, the VLP clusters were in the cytoplasm and appeared separated from P granules (data not shown). Very few or no VLPs were detected in mature oocytes or in early embryonic cells. However, morphologically similar VLPs appeared about midway through embryogenesis in several types of differentiating somatic cells, including hypodermis (skin), muscles, and intestinal cells, where the VLPs often were closely associated with microtubules (Supplemental Figure S1D, S1E, S1F).

The second type of VLP was found in germ cells of four wild strains of *C. elegans* and, surprisingly, the standard laboratory strain, N2 (Table 1). We confirmed the presence of N2 VLPs in (1) worms derived from bleached eggs, indicating that the VLPs are endogenous, (2) in a newly obtained stock of the N2 reference strain from the *Caenorhabditis* Genetics Center, and (3) in a low passage “ancestral” stock of the original N2 isolate (Table 1, http://www.cbs.umn.edu/CGC). Similar to the *C. japonica* VLPs, the *C. elegans* VLPs are about 80 nm in diameter and covered with fine spikes. The interiors of some VLPs contain one or two electron-dense bodies that resemble curved rods and likely correspond to the genetic material (Figure 1F). More commonly, small electron-dense foci are visible in the interior that might represent sectional views of the rods, or distinct stages of maturation or compaction. In contrast to *C. japonica*, the *C. elegans* VLPs were not observed in adult male gonads or in fourth larval stage (L4) hermaphrodite gonads, and were not observed in any embryonic cells (Table 1 and data not shown). The *C. elegans* VLPs were present at much lower abundance than the *C. japonica* VLPs: More than 95% of the *C. elegans* germ cells examined lacked VLPs, and the few positive cells typically contained only one or two VLPs.

We began our analysis on the *C. elegans* VLPs, despite their low abundance, because of the numerous advantages of *C. elegans* for genetic and molecular experiments. To facilitate the analysis, we first asked whether we could increase the number of VLPs through physiological stress (heat, starvation), or by eliminating various pathways (RNAi, cell death) and cell types (coelomocytes) that might have the potential to repress or remove VLPs (Table 2). None of these conditions caused a marked increase in VLPs, although there was a modest and reproducible increase in *alg-1(RNAi); alg-2(ok304)* animals defective in the microRNA pathway (Table 2). We next attempted to use RNAi to identify the source of the VLPs, beginning with the several families of *Cer* elements in the *C. elegans* genome (Table 2). We observed no decrease in VLP numbers with dsRNA directed against the retrotransposons *Cer2*, *Cer3*, *Cer4*, *Cer5*, *Cer6*, or against the endogenous retrovirus *Cer1*3. In contrast, dsRNA against the retrotransposon *Cer1* eliminated all VLPs, strongly suggesting that the VLPs are *Cer1* capsids.

**Table 2:** Electron microscopy of VLPs in *C. elegans* strains.

Genotype or Condition (20–23°C)	Strain	Defective Protein or Pathway	VLP-positive germ cells (%)	Germ cells scored
control	N2		3.9	563
spontaneous steriles	N2		0.0	334
heat shock: 34°C for 45 min, 4 hr recovery	N2		2.4	430
*adr-1(gv6); adr-2(gv42)*	BB4	RNA editing; [70]	0.8	777
*dcr-1(ok247)*	BB1	Dicer; [71]	0.5	743
*rde-1(ne300);ced-1(e1735);ced-3(n717)*	JJ2230	defective in RNAi and cell death pathways; increases titer of viruses in infected cultured cells; [11], [12], [13], [14]	4.0	155
*rde-1(ne300);ced-1(e1735);ced-3(n717)* day 5 adults	JJ2230	defective in RNAi and cell death pathways; increases titer of viruses in infected cultured cells; [11], [12], [13], [14]	0.6	997
*ppw-1(pk2505)*	NL2550	defective in germline RNAi; [72]	0.0	968
*prg-1(n4357); prg-2(n4358)*	MT14769	Piwi proteins; mutants defective in transposon silencing; [73], [74],	0.0	356
*kgb-1(um3);kgb-2(km16)*	KB7	P granule regulation; [75]	0.2	553
*alg-1(RNAi); alg-2(ok304)*	WM53	microRNA pathway; [76]	8.0	510
MAGO12	WM191	mutations in each of 12 Argonaute proteins, RNAi defective; [77]	5.5	604
*unc-119(ed3); arIs37; cdIs32[pcc1::DT-A(E148D); unc-119(+) pmyo2::GFP]*	NP17	no coelomocytes; [78]	6.7	134
*Cer1(RNAi)*	N2	LTR retrotransposon; [8]	0.0	1213
*Cer2(RNAi)*	N2	LTR retrotransposon; [8]	5.9	669
*Cer3(RNAi)*	N2	LTR retrotransposon; [8]	5.5	599
*Cer4(RNAi)*	N2	LTR retrotransposon; [8]	3.8	739
*Cer5(RNAi)*	N2	LTR retrotransposon; [8]	6.3	642
*Cer6(RNAi)*	N2	LTR retrotransposon; [8]	5.1	622
*Cer13(RNAi)*	N2	LTR retrovirus; [8]	2.6	728

**Figure 2:**
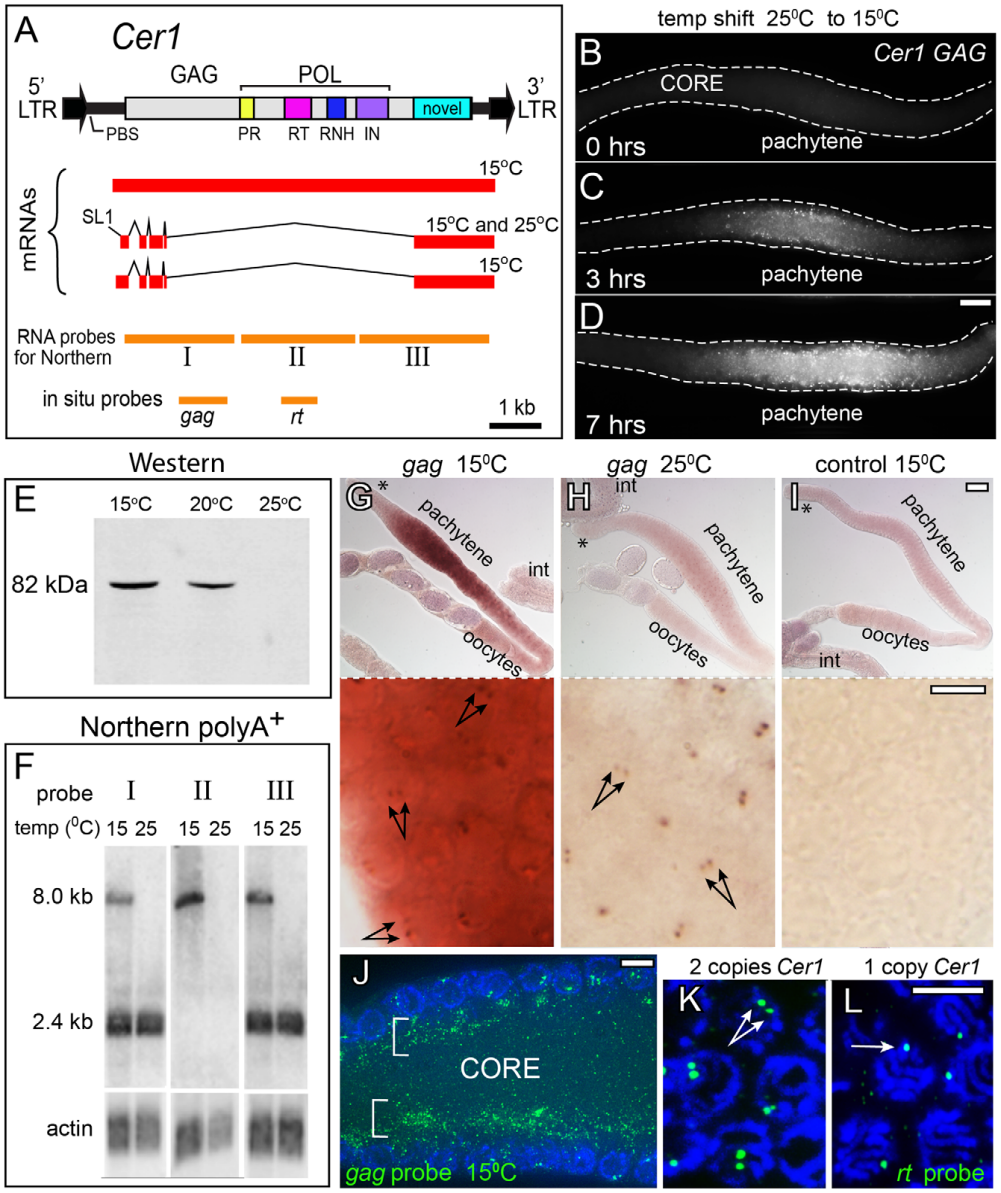
*Cer1* GAG expression is temperature dependent. (A) Diagram of the *Cer1* retrotransposon, mRNA products confirmed by sequencing in this study, and the locations of probes used for Northern and in situ experiments. Colored boxes within POL correspond to homology regions for Protease (PR), Reverse Transcriptase (RT), Ribonuclease H (RNH), and Integrase (IN) as described [30]. mRNAs beginning 5′ to the splice acceptor used for SL1 trans-splicing were demonstrated by RT-PCR using primer pairs KE409F, KE416R and KE409F, KE401BR (Materials and Methods). (B–D) Gonads immunostained with αGAG; gonads were dissected from day 1 adults raised at 25°C, then shifted to 15°C for the times indicated. (E) Western blot of total proteins from adults grown at either 15°C, 20°C, or 25°C; blots were stained with αGAG. (F) Northern blots of poly(A)^+^-selected RNA from adults grown at either 15°C or 25°C; blots were hybridized with probes (I–III) as diagrammed in panel A. Normalization relative to a control actin probe showed that the difference in signal between 15°C and 25°C is at least tenfold for the 8 kb band. (G–I) RNA in situ hybridization (alkaline phosphatase detection) of gonads and intestines (int) from adults cultured at the indicated temperatures, using probes for sense or antisense (control) *gag*-containing mRNA; each asterisk indicates the distal, mitotic region of the gonad. Bottom panels are higher magnifications of the same gonads showing *gag*-containing RNA concentrated in paired, nuclear dots (double arrows). (J) Fluorescence in situ hybridization (FISH) of a 15°C gonad using the *gag*-specific probe (green); the image shows a longitudinal, optical section through the gonad core. Note that RNA is concentrated in the superapical region of the core (brackets), and in paired dots within nuclei (blue, DAPI). (K–L) High magnification of FISH with the *rt*-specific probe; images show two nuclear dots in wild-type germ cells (panel K, two copies of *Cer1*) and one dot in nuclei from N2/CB4856 heterozygotes (panel L, one copy of *Cer1*); control CB4856 homozygotes which lack *Cer1* show no staining (data not shown). Scale bars: B–D (20 µm); G–I (20 µm), inserts (5 µm); J–L(5 µm).

*Cer1* is an 8.8 kb LTR retrotransposon in the Gypsy/Ty3 family of retroviruses/retrotransposons (Figure 2A) [29]. *Cer1* has a single, long open reading frame with the potential to encode a GAG- and POL-containing polyprotein [30]. The N2, laboratory strain of *C. elegans* contains one, apparently intact, copy of *Cer1*, and three LTR fragments [8]. The *Cer1* insertion in N2 disrupts *plg-1*, a mucin-like gene that is required for the formation of the copulatory plug in mated adults; wild *C. elegans* strains are polymorphic for this insertion [31]. The 5′ and 3′ LTRs of *Cer1* are identical, which can indicate a recent insertion as both LTRs are generated from a single template during retrotransposon replication [32]. However, there is no evidence that *Cer1* remains active in N2: No spontaneous mutations have been shown to result from the transposition of *Cer1*, or of any other *Cer* element [9], [10], and no mRNAs that can encode essential GAG or RT proteins from *Cer1* have been identified in any of several *C. elegans* cDNA and EST (Expressed Sequence Tag) projects (compiled at the Wormbase web site, http://www.wormbase.org, release WS227, 2011). Finally, *Cer1* was reported to lack an obvious Primer Binding Site (PBS) [8]. A PBS is critical for replication, and typically is found near the 5′ LTR; the complementary 3′ end of a specific host tRNA binds the PBS to prime minus-strand DNA synthesis. However, in a search of the tRNA database GtRNAdb [33] we found that the sequence TGGGGGCCGAACCG, which is directly adjacent to the *Cer1* 5′LTR, is complementary to the 3′ end of *C. elegans* tRNA-Pro(TGG) (Supplemental Figure S2). The identical sequence was conserved in a group of *C. japonica* LTR retrotransposons we identified independently in best reciprocal BLAST searches with the *Cer1* GAG sequence (Supplemental Figure S2). In a search of the *C. elegans* genome for additional copies of the presumptive PBS, we discovered LTRs and protein coding fragments from a larger, now extinct family of *Cer1*-like retrotransposons (Supplemental Figure S2). Although those LTRs and the *C. japonica* LTRs are highly diverged from *Cer1*, each contains one or more predicted binding sites for the *C. elegans* E2F transcription factor, EFL-1 (Supplemental Figure S2) [34]. In preliminary studies, we found that a transgene containing the 5′LTR of *Cer1* and partial coding sequences linked to GFP (Green Fluorescent Protein) was expressed in germ cells, but was expressed in somatic cells instead of germ cells when the predicted E2F binding site GGCGCGAA was mutated to GGGCCGAA (our unpublished results).

### Temperature dependence of *Cer1* GAG expression

Because GAG proteins are the main structural components of retroviral and retrotransposon capsids, we generated a monoclonal antibody (αGAG) against the predicted *Cer1* GAG protein. αGAG stained small, uniform puncta in N2 gonads (Figure 2B–D), but showed no staining in *Cer1*(*RNAi*) gonads, or in gonads from the CB4856 Hawaiian strain of *C. elegans* that lacks the *Cer1* sequence [31] and that lacks VLPs by electron microscopy (Table 1, Table 3, and data not shown). Similarly, αGAG did not stain gonads from N2 adult males or L4 hermaphrodites that lack VLPs by electron microscopy (Table 3). On Western blots of total worm proteins, αGAG stained a single band of about 82 kDa, compared to a predicted GAG size of 83–86 kDa (Figure 2E). This protein is much smaller than the size of the predicted *Cer1* polyprotein, suggesting that the polyprotein is cleaved after synthesis. Unexpectedly, in the course of these experiments we discovered that GAG expression was strongly dependent on culture temperature and adult age. Worms grown at 25°C showed no detectable GAG expression by immunostaining or by Western analysis (Figure 2B, 2E and Table 3). GAG was expressed at low and variable levels in adults cultured at 20–23°C, but was expressed at much higher levels in 15°C adults. Moreover, GAG expression could be either induced, or repressed, by shifting adult animals between 15°C (ON) and 25°C (OFF; Figure 2B–D, Table 3). Because our initial survey of *Caenorhabditis* wild strains, and of N2-derived mutant strains, was performed on 1 day old (day 1) adults raised at standard culture temperatures of 20–23°C, we repeated electron microscopy on day 1 and day 3 N2 adults grown at 15°C. We found that VLPs (hereafter designated as *Cer1* capsids) were much more abundant at 15°C than observed previously at higher temperatures (see below). We have not yet been able to determine if the *C. japonica* VLPs have similar temperature sensitivity: Abundant VLPs were observed in four separate *C. japonica* cultures grown over a 4 month interval in the first stages of this study. However, we observed only a few VLPs in more recent preparations, suggesting that expression is subject to silencing.

**Table 3:** *Cer1* GAG particles detected by immunostaining.

Genotype, strain, or condition	Defective protein or pathway	Culture temp (°C)	Stage	Shift temp (°C)	Duration of shift (hrs)	GAG particles (range)	Gonads scored
CB4856		15	A			−	>>100
CB4856		25	A			−	>>100
WT		15	A			+, +++	>>100
WT		20	A			−, +	70
WT		25	A			−	>>100
WT		15	L4			−	23
WT		20	L4			−	15
WT		25	L4			−	44
WT male		15	A			−	18
WT male		25	A			−	18
WT		15	L4	25	21	−	62
WT		15	A	25	21	−	41
WT		15	A	25	10	+	49
WT		25	A	15	10	+, +++	50
*fem-1(hc15ts)*	spermatogenesis	25	L4	15	24	+, +++	11
*hsf-1(sy441)*	heat shock [35]	15	A			+, ++	14
*hsf-1(sy441)*	heat shock [35]	15	L4	25	21	−	15
*hsf-1(sy441)*	heat shock [35]	15	A	25	21	−	21
WT, 350 mM NaCl	osmotic stress [36]	15	A			+, +++	12
*dhc-1(or352ts)*	dynein [41]	15	A			−(50%), +(30%), ++(20%)	20
*dhc-1(or283ts)*	dynein [41]	15	A			−(26%), +(42%), ++(32%)	19
*dhc-1(or283ts)*	dynein [41]	15	A	25	1	−(40%), +(28%), ++(32%)	25
*dhc-1(or283ts)*	dynein [41]	15	A	25	2	−(52%), +(17%), ++(31%)	23
*dhc-1(or283ts)*	dynein [41]	15	A	25	21	−(98%) +(2%)	41
*alg-2(ok304); alg-1(RNAi)*	microRNA pathway, [76]	25	A			−	26
*ergo-1(tm1860)*	endogenous siRNAs, [77]	20	A			+	39
*ergo-1(tm1860)*	endogenous siRNAs, [77]	25	A			−	24
WM126	mutations in 6 Argonautes, [77]	25	A			−	26
WM191	mutations in 12 Argonautes, [77]	20	L4	25	25	−	>20
WM191	mutations in 12 Argonautes, [77]	25	A			−	>20

Culture temperatures of 27°C and above are stressful or lethal for *C. elegans*, and even 25°C can activate variable, low-level expression of some heat shock reporters in some somatic tissues (our unpublished results). To determine whether the lack of *Cer1* expression at 25°C resulted from activation of the heat shock pathway, we examined *hsf-1(sy441)* mutants defective in the heat shock regulator HSF-1 [35]. Similar to wild-type adults, the *hsf-1(sy441)* mutants expressed GAG at 15°C, but not 25°C. Conversely, osmotic stress from culture media containing 350 mM NaCl is sufficient to activate a heat shock response at low temperature (see [36] and references therein), but was unable to repress GAG expression at 15°C (Table 3). To determine whether temperature affected *Cer1* mRNA expression, Northern analysis was performed on poly(A)-selected RNA from adult hermaphrodites cultured at either 15°C or 25°C, using probes specific for three regions spanning the *Cer1* coding region (Figure 2A and 2F). These results showed that a *Cer1* transcript of about 2.4 kb is produced at both 15°C and 25°C, but that a large transcript of about 8 kb is detectable only at 15°C. We next sequenced RNAs that were amplified by RT-PCR using primers for various *Cer1*-specific sequences, and an additional primer specific for SL1 (Spliced Leader 1); SL1 is a 22 nt trans-spliced leader sequence found on about 60% of *C. elegans* mRNAs (Figure 2A and Materials and Methods) [37]. We found that the 8 and 2.4 kb mRNAs correspond to the sequences diagrammed in Figure 2A; the 2.4 kb message is spliced and is identical to the sole *Cer1* mRNA identified previously in *C. elegans* EST and cDNA projects (Wormbase web site, http://www.wormbase.org, release WS227, 2011). These results show that *Cer1* capsids are not detectable at 25°C because the 8 kb mRNA that encodes the GAG and POL proteins is not detectable at that temperature.

The spliced 2.4 kb mRNA can encode a novel, approximately 60 kDa protein that includes discontinuous, but in-frame, peptide sequences shared with the N-terminus of GAG (Figure 2A). The spliced mRNA utilizes *Cer1* sequences that conform to consensus acceptor and donor intron splice sites for *C. elegans* (Supplemental Figure S3) [38]. This splicing pattern is likely to be a general feature of the *Cer1* family, as *Cer1*-related retrotransposons in both *C. remanei* and *C. japonica* contain consensus splice acceptor and donor sequences at similar positions (our unpublished results). We detected the spliced mRNA with, and without, the SL1 leader at both 15°C and 25°C (Figure 2A, 2F and data not shown). In contrast, the 8 kb message did not appear to be spliced, and was not trans-spliced at the site utilized by SL1; it could not be amplified with an SL1-specific primer, but was amplified with primers either 5′ or 3′ of the SL1 site (see legend to Figure 2A). Thus, capsid production at 15°C requires that multiple, consensus splice sites within *Cer1* are skipped in order to generate the 8 kb mRNA that encodes GAG and POL.

We next performed in situ hybridization on fixed gonads to examine the spatial and temporal expression of the 8 kb mRNA, using probes specific for either *rt* or *gag* (diagrammed in Figure 2A). At 15°C, *gag*-containing RNA was first detectable in the early pachytene region of the gonad, but was not detected in somatic tissues such as the intestine (Figure 2G, 2I). The gonad expression correlates with the onset of GAG immunostaining (Figure 2D), with the first appearance of VLPs by electron microscopy (Supplemental Figure S1G), and is similar to the onset of EFL-1/E2F expression [39]. Fluorescence In Situ Hybridization (FISH) further showed that the RNA was enriched in the superapical zone of the gonad core, with relatively little RNA in the cytoplasm surrounding germ nuclei (Figure 2J). Interestingly, both alkaline phosphatase and FISH detection protocols showed *gag*- and *rt*-containing RNA concentrated in a pair of nuclear dots (Figure 2G, 2J and Figure 2K). No nuclear dots were observed in CB4856 worms that lack *Cer1*, and only one nuclear dot was observed in N2/CB4856 heterozygotes that have one copy of *Cer1* (Figure 2L and data not shown). These results suggest that the double dots in N2 represent RNA at or near the site of *Cer1* insertion on the paired, homologous chromosomes. Similar nuclear dots were detected in N2 gonads at 25°C that otherwise lack the *gag*-containing mRNA (Figure 2H). These results suggest that *gag*-containing RNA is transcribed at 25°C, but fails to accumulate in the cytoplasm. Finally, we wanted to determine whether the temperature-dependence of GAG expression was unique to the N2 copy of *Cer1*, or was a more general characteristic of *Cer1* retrotransposons. Using available data on the pattern of *Cer1* insertion in various wild *C. elegans* strains [31], we immunostained 15 wild isolates that have the N2 pattern of *Cer1* insertion in the *plg*-1 gene, and 15 strains with *Cer1* inserted elsewhere in the genome. 21 of the 30 strains contained immunoreactive GAG particles at 15°C that closely resembled the N2 staining pattern, but none contained GAG particles at 25°C (Table 4).

**Table 4:** *Cer1* GAG particles in *C. elegans* wild strains.

Strains	GAG Particles		*Cer1* a	*Cer1 insertion in plg-1* a
	15°C	25°C		
AB1, CB3191, CB4507, CB4555, CB4851, CB4932, DH424, EG4346, JU311, JU440, JU361, JU394, LSJ1, N2, PX176, TR388	yes	no	present	*yes*
ED3040, JU345, JU395, JU1171, KR314, PB306	yes	no	present	no
CB4854, CB4856, ED3072, JU262, JU263, JU322, JU360, JU362, JU363, JU438, JU1088, MY1, PB303, PX174, RC301	no	no	absent	NA
AB2, CB4853, CB4855, CB4858, ED3005, ED3077, JU258, MY2, MY14	no	no	present	no

a Data from [31].

### *Cer1* capsid localization in germ cells

**Figure 3:**
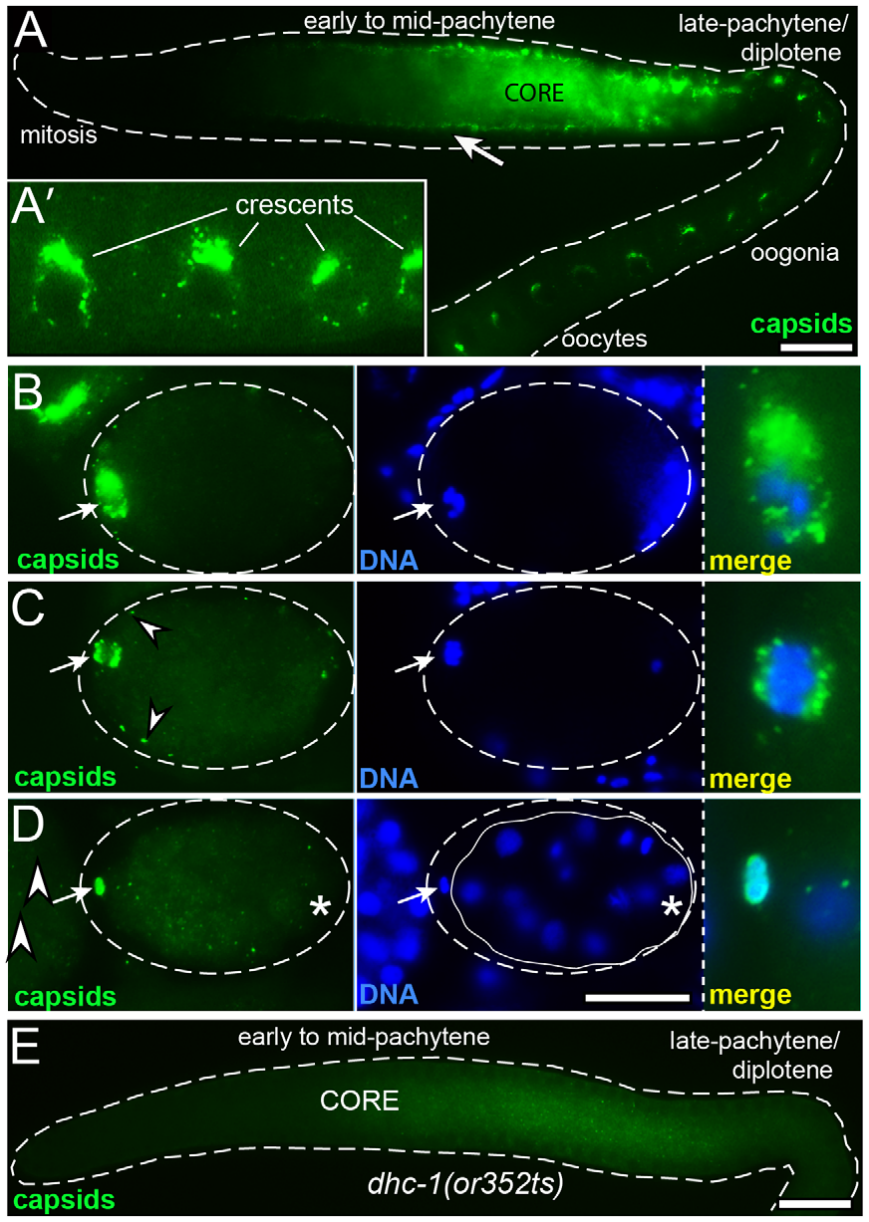
*Cer1* capsids show stage-specific changes in localization. (A) Low magnification, wide-field image of a gonad from a 15°C adult, immunostained with αGAG to detect *Cer1* capsids (green). Note that capsids in the mid-pachytene region are enriched along the superapical zone of the core (compare with Figure 2J), rather than within germ cells at the periphery (arrow). (A′) High magnification showing the crescent-shaped aggregates of capsids by oogonia nuclei. (B,C) Newly fertilized eggs showing the maternal chromosomes at diakinesis (B), and at metaphase of the meiosis I division (C). Arrows point to concentrations of capsids associated with the meiosis I spindle, and arrowheads indicate small foci of capsids at the periphery. The examples shown for these images contain unusually high numbers of capsids; most eggs show the same localization pattern but with fewer capsids, and many fertilized eggs contain no detectable capsids. (D) 28-cell stage embryo; the arrow points to capsids within the first polar body. The asterisk is below the germ cell precursor, showing that capsids do not concentrate in this cell. The eggshell is outlined in panels B–C (dashed line), and the cellular boundary of the 28-cell embryo is indicated in panel D (white line). (E) Capsid localization in a *dhc-1*/dynein mutant; this gonad was scored as “+” in Table 3. Scale bars: A–E (20 µm).

**Figure 4:**
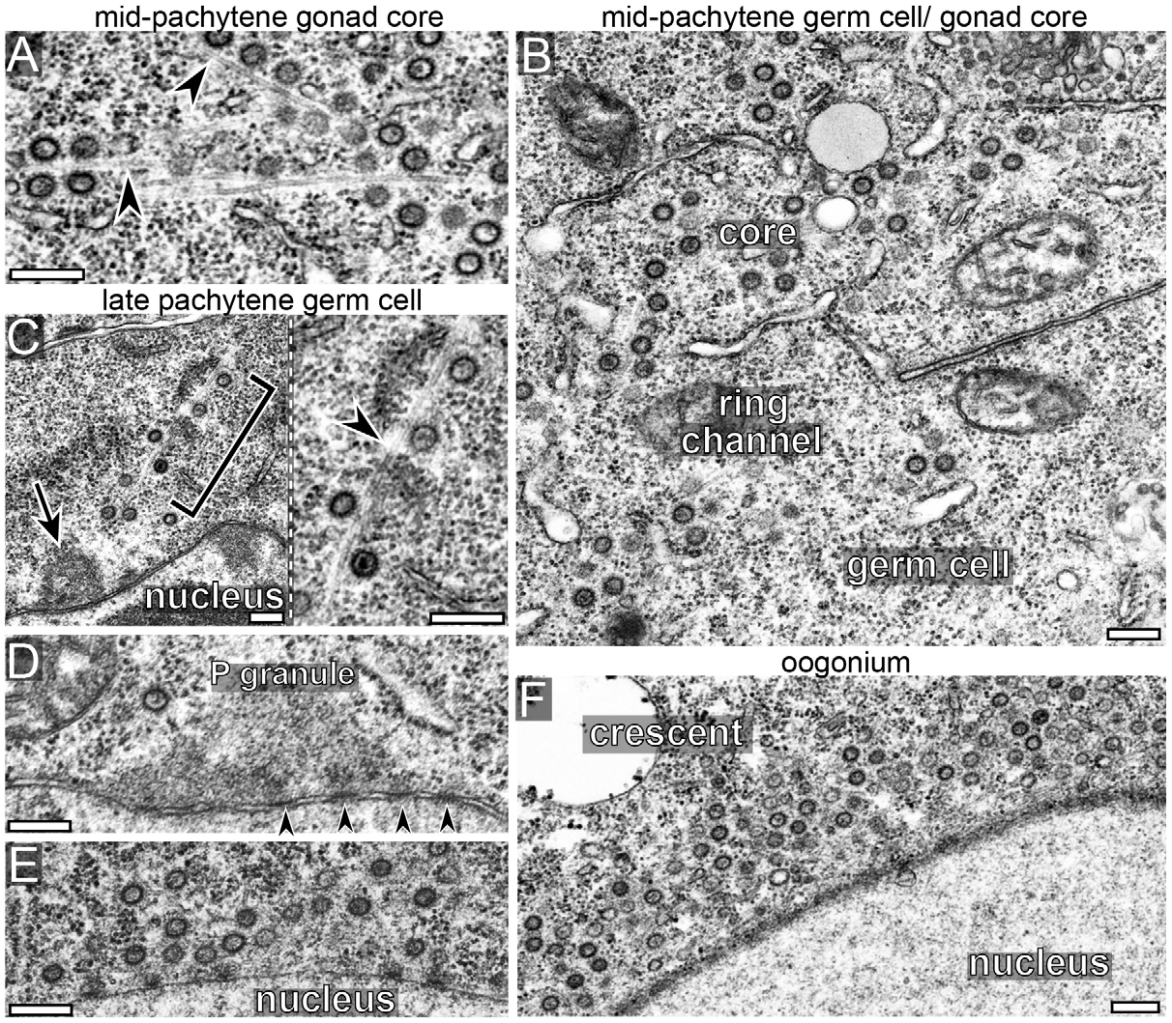
*Cer1* capsids localize on microtubules. (A–F) Electron microscopy of gonads from 15°C adults; regions of the gonad are as indicated at the top of the panels. Capsids localize predominantly to the core in the mid-pachytene region, but concentrate near nuclei in late-pachytene germ cells (C–E) and in oogonia (F). Note that many capsids localize with microtubules (arrowheads) both in the core (panel A) and in germ cells (panel C). P granules are visible in panels C (arrow) and in panel D; arrowheads in panel D indicate examples of nuclear pores. (E) Cluster of capsids near the nucleus of an early oogonium. (F) A “crescent” of capsids by an oogonium nucleus; there are at least 64 capsids and numerous microtubules visible at higher magnifications of this single thin section (data not shown). Scale bars: A–F (0.2 µm).

**Figure 5:**
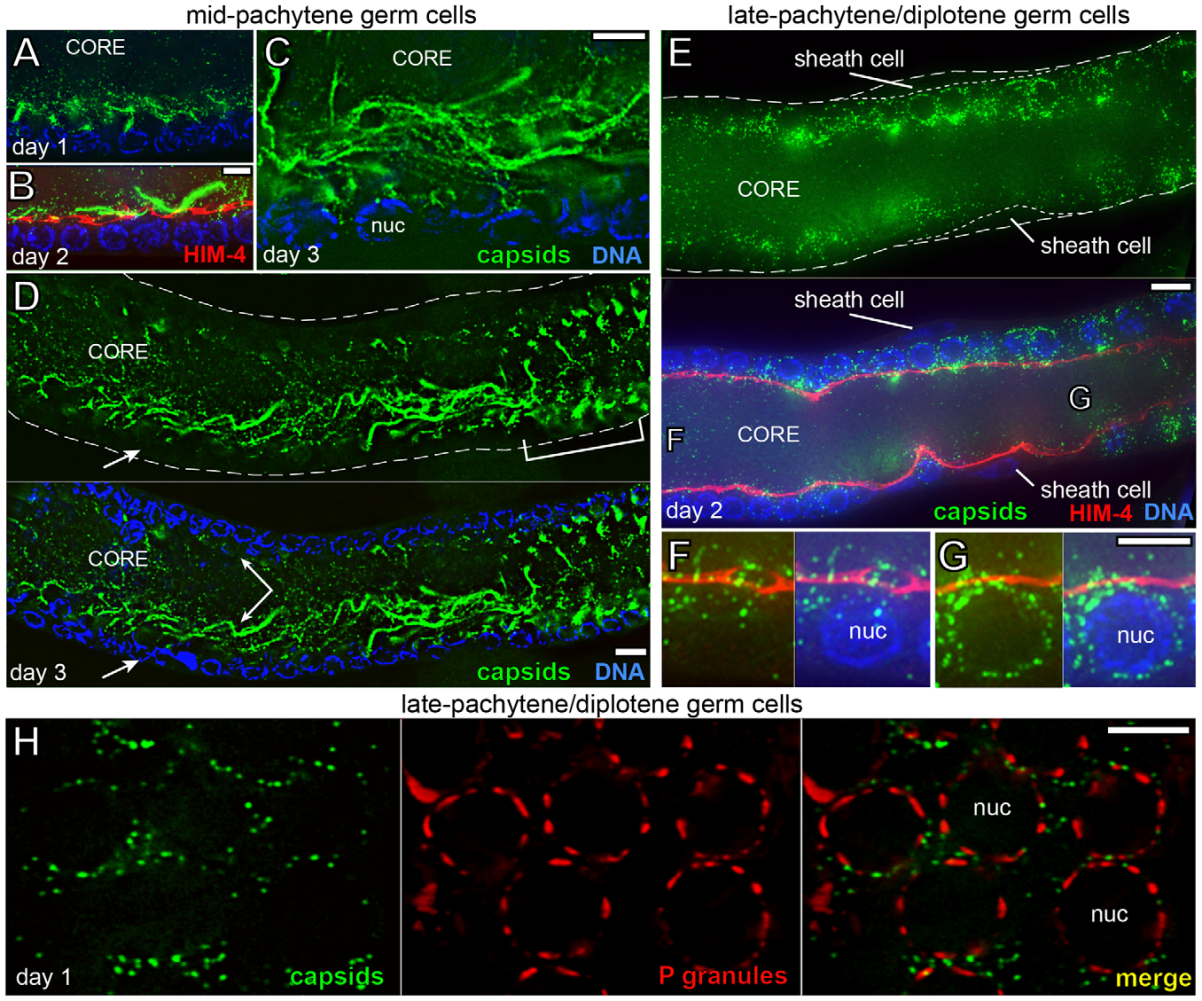
Capsids accumulate in the mid-pachytene gonad core as adults age at 15°C. (A–D) Longitudinal, optical sections through gonads showing increased accumulation of capsids (green) with adult age, as indicated. The gonad in panel B is immunostained for HIM-4/hemicentin (red) to visualize the apical membrane (see Figure 1B). Note that capsids localize predominately in the core, outside the ring channel and away from nuclei (blue, DAPI). (C) Capsids grouped in wavy lines and tangles of lines in the mid-pachytene region of a day 3 adult. (D) Low magnification of the mid-pachytene region. Note variation in capsid abundance between the two sides of the gonad core (double-headed arrow), and that the wavy lines of capsids disappear as germ cells move proximally into late pachytene (bracketed region). (E–G) The late-pachytene/diplotene region, stained and imaged as for panel B; two of the somatic sheath cells that surround the gonad are visible in this image. Germ cells from regions F and G are shown at high magnification, oriented as in Figure 1B. An optical rotation of a similar region of the gonad is shown in Supplemental Video S1. (H) Optical section through germ nuclei in the late-pachytene/diplotene region showing capsids (green) and P granules (red, αPGL-1). Note that capsids localize close to the nuclear envelope, but most are not directly on, or within, P granules. Electron micrographs of capsids within P granules are shown in Supplemental Figure S1H. Scale bars: A–C, F–H (5 µm), D–E (10 µm).

Essentially all of the immunostaining results for GAG-containing particles described here correlate with *Cer1* capsid localization determined by electron microscopy. Therefore, we refer to the immunostained, GAG-containing particles simply as *Cer1* capsids. In support of this designation, biochemical studies to be described elsewhere show that the GAG protein detected on Western blots of worm extracts purifies with a sedimentation profile typical of viruses or capsids (our unpublished results). In overview, *Cer1* capsids first appear in early- to mid-pachytene, where they localize almost exclusively in the superapical region of the gonad core, outside of the germ cell proper and away from nuclei (Figure 3A). By electron microscopy, capsids are present in small groups that are closely associated with rough endoplasmic reticulum (Supplemental Figure S1G), but capsids later appear more dispersed and associated with microtubules (Figure 4A, B). Capsids remain largely confined to the gonad core throughout the mid-pachytene region, but during late pachytene/diplotene most capsids accumulate around, or at the base of, germ nuclei (Figure 3A and Figure 4C–4E; see also Figure 5E).

In oogonia and early oocytes, most capsids are concentrated in large, crescent-shaped aggregates that are adjacent to the nuclear envelope (Figure 3A and Figure 4F). In mature oocytes, capsids disperse throughout the cytoplasm and most disappear (data not shown). The small population of capsids that persist in newly fertilized embryos exhibit two additional localization patterns: When fertilization initiates the first of two meiotic divisions of the maternal chromosomes, capsids localize equally to both poles of the meiosis I spindle (Figure 3B, 3C). This spindle rotates perpendicular to the anterior surface of the embryo, and during division half of the maternal chromosomes are extruded from of the embryo as the first polar body; capsids at the anterior spindle pole are similarly extruded into the polar body (Figure 3D). During these stages, additional capsids localize in small foci distributed across the periphery of the embryo (arrowheads in Figure 3C). This peripheral localization occurs at about the same time as much larger cortical granules move to the surface and are exocytosed to form an extra-embryonic layer [40]. The peripheral capsids are not visible at later stages, suggesting that some of these are externalized and/or degraded. Very few or no capsids persist into cleavage-stage embryos, and any remaining capsids show no enrichment in the embryonic germ cell precursors (asterisk in Figure 3D). Previous studies showed that cytoplasmic dynein and dynein-associated proteins localize to both poles of the meiosis I spindle and to foci at the periphery of the newly fertilized embryo [41], similar to the localization of *Cer1* capsids. We found that each of two temperature-sensitive alleles of *dhc-1*/dynein resulted in variable and markedly reduced levels of capsid staining throughout the gonad, even at the permissive temperature for both mutations (Figure 3E, quantified in Table 3). We do not yet know from biochemical experiments whether dynein is directly associated with capsids, but we did not observe significant colocalization of DHC-1/dynein and *Cer1* capsids in gonads by immunostaining (data not shown).

### Capsids accumulate progressively with adult age

We found that capsid accumulation requires oogenesis but not spermatogenesis (Table 3), and is strongly dependent on adult age. For the latter analysis, we cultured hermaphrodites in the constant presence of males, as oogenesis arrests in the absence of sperm. By the second day of adulthood (day 2 adults) and increasing thereafter, many gonads contain huge numbers of capsids organized into linear arrays that resemble wavy lines, or tangles of wavy lines (Figure 5A–5D, and Supplemental Figure S4A, S4B). Costaining for HIM-4/hemicentin, which localizes by the apical membrane [42], showed that the tips of the wavy lines occasionally and partially enter germ cells through the ring channels (Figure 5B). However, the vast majority of capsids localize in the core, outside of germ cells and away from nuclei, similar to capsids in younger adult gonads (Figure 4B and Figure 5A–5D). The abundance of capsids, and the presence and size of the lines and tangles, varies considerably for germ cells that are at the same developmental stage, but at different positions in the gonad core (double arrow in Figure 5D).

Both young and older adults show a marked shift in capsid distribution as germ cells begin to exit pachytene, in the pachytene/diplotene region: Nearly all of the lines and tangles of capsids disappear from the core, and large numbers of capsids redistribute into germ cells and around nuclei (Figure 4E, Figure 5E–5G, and Supplemental Video S1). In normal development, the late pachytene/diplotene nuclei enlarge and add new nuclear pores that are not covered by P granules; all P granules are eventually shed from germ nuclei during subsequent oogenesis [20], [21]. Thus, capsids in both young and older adults only accumulate around germ nuclei with available, P granule-free nuclear pores. Electron microscopy, and co-staining for capsids and P granules, showed that most capsids do not accumulate directly on P granules (Figure 4D, and Figure 5H), although a few capsids are found within P granules at this stage (Supplemental Figure S1H). Instead, most capsids are located near and between P granules, and appear directly associated with microtubules (Figure 4C, 4E and Figure 5H).

### Changes in *Cer1* capsid localization parallel changes in stable microtubules

**Figure 6:**
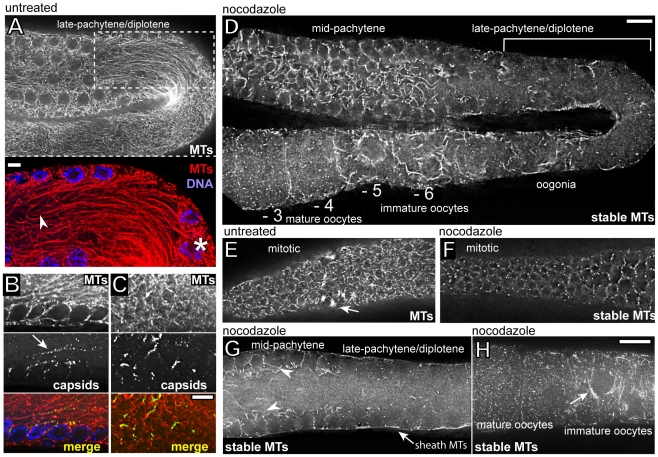
A subset of microtubules in the mid-pachytene region are inhibitor-resistant, or stable. (A) Optical section through the superapical plane of an untreated day 1 adult gonad showing the dense network of long microtubules (see Figure 1A for section orientation). The inset shows a high magnification of the dashed box, with an arrowhead marking the end of a single microtubule traced from the indicated germ cell (asterisk). (B,C) Optical sections through the central planes of untreated, day 1 adult gonads; the gonads are immunostained for both microtubules and *Cer1* capsids. Note that capsids are concentrated in linear or irregular shapes, while the microtubules are distributed uniformly. (D) Optical section through the superapical plane of a day 1 adult gonad treated with nocodazole; the bracketed region is the same late-pachytene/diplotene region, and the same optical plane, as for the untreated gonad in panel A. Note that most microtubules have disappeared from this region after nocodazole treatment, in contrast to the numerous “stable” microtubules that remain in the mid-pachytene region. (E,F) Microtubules in the distal, mitotic regions of gonads before (E) and after (F) nocodazole treatment. The arrow in panel E indicates an example of a mitotic spindle. Most of the brightest spots of tubulin visible in panel F co-localize with centrosomes (data not shown). (G) Optical section through the central plane of a nocodazole-treated gonad. This sectional view combined with that in panel D illustrates that most of the stable microtubules in the mid-pachytene region are in the superapical zone of the core (arrowheads). Note that most of the microtubules in the late-pachytene/diplotene germ cells have depolymerized; stable microtubules that appear at the periphery of the gonad (arrow) are outside of germ cells and within the thin cell bodies of somatic sheath cells. (H) Stable microtubules in nocodazole-treated oocytes; the oocytes advance in age right to left (see Figure 1A). Note the focus of stable microtubules (arrow) near the oocyte nucleus, and the abrupt disappearance of all stable microtubules in the more mature oocyte to the left; compare with oocyte progression panel D, where numbers indicate oocyte position relative to ovulation. Scale bars: A–C (5 µm), D–H (10 µm).

Several observations suggested that microtubules are involved in capsid localization. First, we found that about 30% of capsids scored in randomly selected electron micrographs appeared to contact one or more microtubules (n = 180/560 capsids, Figure 4A, 4C). As microtubules that don't present clear cross-sectional or longitudinal profiles in micrographs are difficult to score, this percentage likely underestimates the association. Second, aggregates of large numbers of capsids and microtubules were visible in electron micrographs of day 3 and older adults that resembled the wavy lines and tangles detected by immunostaining (Supplemental Figure S4A, S4B). Finally, the localization of capsids in newly fertilized embryos closely resembles that of cytoplasmic dynein, as described above. Despite the above correlations, the distribution of most capsids in immunostained gonads bears little resemblance to the distribution of microtubules: Microtubules are highly abundant throughout the gonad (Figure 6A), while capsids are concentrated in much smaller regions (Figure 6B, 6C).

To determine whether capsid localization was dependent on microtubules, we treated gonads with the inhibitors nocodazole or colcemid. Cytoplasm normally flows proximally through the gonad core, transporting materials toward and into enlarging oocytes, but bypasses the late-pachytene/diplotene germ cells where capsids accumulate [27]. Flow is actin-, but not microtubule- dependent, and can carry transport large, inert beads a distance of nearly half the length of the gonad in only 25–30 minutes [27]. Thus, we anticipated that if microtubules normally anchor the capsids against flow, the capsids would flush proximally in inhibitor-treated gonads. Instead, the inhibitors caused little if any change in the distribution of capsids throughout the early- to mid-pachytene region, including the wavy lines and tangles of capsids present in older adults (data not shown and see below).

**Figure 7:**
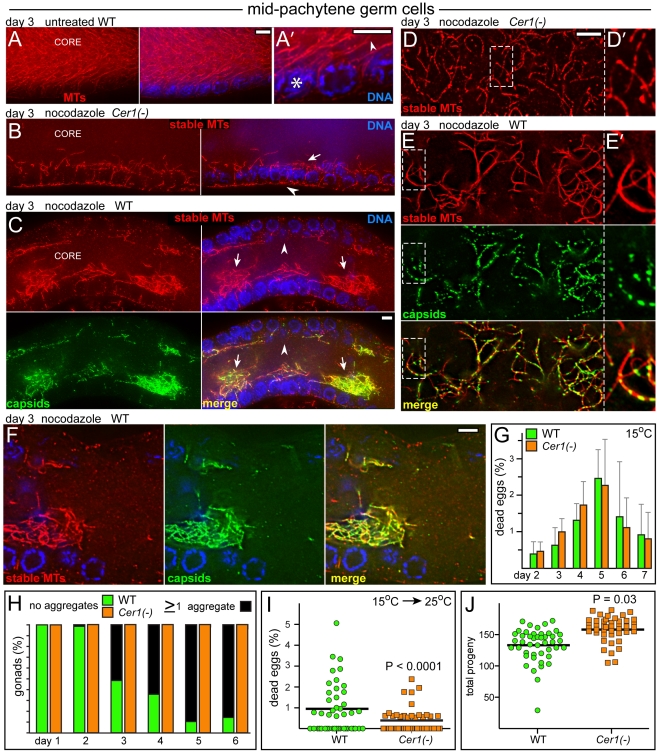
*Cer1* capsids accumulate on stable microtubules in the mid-pachytene region. (A–F) All images are of the mid-pachytene region of day 3 adult gonads. Panels A–C and F show central optical planes, and panels D,E show superapical optical planes. (A) Untreated wild-type gonad, showing the dense population of total microtubules in the core; panel A′ indicates a basket microtubule (arrowhead) traced back to the germ cell of origin (asterisk). (B) Stable microtubules in a nocodazole-treated, Cer1(-) gonad. Most of the core microtubules have depolymerized, and the stable microtubules that remain are enriched in the superapical zone (arrow); the arrowhead points to stable microtubules in sheath cells. Stable microtubules in a nocodazole-treated, wild-type gonad. Note the multiple aggregates of stable microtubules (arrows) and the neighboring regions devoid of microtubules (arrowhead). Note also that capsids are strongly concentrated in the microtubule aggregates (see also panel D below). (D–E) Images of stable microtubules as in panels B and C, respectively, but viewed from a superapical plane. Note the thick and irregular staining of the wild-type stable microtubules, and their association with *Cer1* capsids. Panels at right are high magnifications of the dashed, boxed regions at left. (F) High magnification image of an aggregate from the gonad shown in panel C. Note the abrupt loss of stable microtubules, and most capsids, to the right of the panel as germ cells exit pachytene (see also Figure 8B). (G) Quantitation showing mean and standard deviation in the number of dead eggs laid by wild-type and *Cer1(-)* adults grown for the indicated number of days at 15°C. This analysis was performed on 20–25 separate pools of 5 adults each that were cultured in the constant presence of males to maintain ovulation. Brood sizes remained comparable up to day 5 (WT = 437 +/− 57 total progeny, *Cer1(-)* = 442 +/− 83), after which some adults in both sets stopped laying eggs. (H) Percentage of gonads without aggregates of stable microtubules in wild-type (green) and *Cer1(-)* (orange) animals as a function of adult age. Note that nearly all wild-type gonads contain 1 or more aggregates by day 5. (I, J) Analysis of dead eggs and total progeny produced within 24 hours after shifting 3 day adults from 15°C to 25°C. Dead eggs were scored in panel I only if they were approximately normal size. The wild-type adults also produced significantly more small, “partial” eggs than *Cer1(-)* [WT = 2.9%+/− 0.4 partial eggs, *Cer1(-)* = 1.6% +/− 0.2; P<.0001]. Scale bars: A–F (5 µm).

The nocodazole concentration used for those experiments (10 µg/ml; 1 hr) was the same used in previous studies on gonad microtubules [28]. However, we discovered that a large subset of microtubules was not depolymerized by that treatment, or even higher doses of nocodazole (40 µg/ml; 2 hrs). For our subsequent analysis, we chose a standard dose of 10 µg/ml nocodazole for 30 minutes, and we refer to microtubules that resist depolymerization under these conditions as stable microtubules. We found that this dose of nocodazole effectively depolymerized dynamic microtubules in the spindles of mitotic germ cells in the distal gonad (Figure 6E, 6F), and depolymerized dynamic microtubules in the most mature oocytes (Figure 6D, 6H) [43]. In contrast, large numbers of stable microtubules persisted in the early- to mid-pachytene region, where they were concentrated along the superapical zone of the gonad core (Figure 6D, 6G). A subset of the stable microtubules could be traced into germ cells, indicating that some of these are basket microtubules (Figure 1B, Figure 6G, and data not shown). *Cer1* was not the source of microtubule stability, as gonads in each of the following types of animals that lack *Cer1* capsids contained stable microtubules: L4 hermaphrodites, adult males, adult hermaphrodites at 25°C, and adult hermaphrodites treated with *Cer1*(dsRNA) at 15°C (n = 30–75 gonads scored for each, data not shown). In addition, stable microtubules were observed in the gonads of a hybrid strain we constructed where the intact *plg-1* gene from CB4856 was introgressed into N2 to replace *Cer1* (see Figure 7B below); we refer to these N2-derived animals that lack *Cer1* as *Cer1(-)*.

To determine the relationship with *Cer1* capsids, we examined stable microtubules (after nocodazole treatment) in wild-type and *Cer1(-)* animals that were grown as adults for 1 to 6 days at 15°C in the presence of males. We found a striking correspondence between the localization of capsids and the stable subset of microtubules in adults at all ages, and in all regions of the gonad. The following describes our results for the mid-pachytene region, where nearly all capsids localize to the core (Figure 7A–7E). In control experiments, the pattern of total microtubules (stable plus labile) in untreated, day 1–6 wild-type gonads appeared identical to the pattern in untreated, age-matched *Cer1(-)* gonads (Figure 6B, Figure 7A, and data not shown). The pattern of stable microtubules in day 1–2 wild-type gonads (Figure 6G) closely resembled that in day 1–6 *Cer1(-)* gonads (Figure 7B, and data not shown); in both types of gonads, the stable microtubules were enriched in a uniform layer along the superapical zone of the core. Beginning on day 3, however, the wild-type gonads often contained aggregates of stable microtubules (Figure 7C, 7F). The percentage of gonads with aggregates, and the number of aggregates per gonad, both increased with adult age, such that most wild-type gonads contained at least one aggregate by day 6 (Figure 7H). Remarkably, essentially all capsids throughout the mid-pachytene region were localized on stable microtubules, and capsids were particularly concentrated in the aggregates (Figure 7C, 7F). While the stable microtubules in *Cer1(-)* gonads had a relatively uniform thickness (Fig. 7D), the capsid-bearing stable microtubules in wild-type gonads varied markedly in thickness, suggesting they were bundled (Fig. 7E). Together, these several results show that stable microtubules exist independent of *Cer1* capsids, but that capsids appear to cause an age-dependent aggregation of the stable microtubules. The microtubule aggregates are not apparent in untreated wild-type gonads immunostained for tubulin, presumably because of the high density of total microtubules. However, the presence of microtubule aggregates in untreated gonads is confirmed directly by electron microscopy (Supplemental Figure S4A, S4B), and indirectly by the localization of capsids (Figure 5C, 5D).

The largest aggregates of stable microtubules and capsids appear to bridge across the entire diameter of the gonad core, and are often adjacent to regions nearly devoid of stable microtubules (arrowhead in Figure 7C). Thus, the aggregates might grow by stripping stable microtubules from neighboring regions. If the function of the stable microtubules is to maintain gonad architecture, as we consider likely, we wondered whether the aggregates might impact fertility. We compared age-matched wild-type and *Cer1(-)* adults over each of 7 days at 15°C, but did not observe a significant difference in the number of dead eggs laid (n>20,000 total eggs for each strain; Figure 7G). We next cultured both strains at 15°C until day 3, to pre-load the wild-type gonads with capsids, then shifted the adults to 25°C and examined the eggs laid over the next 24 hour period. The wild-type, shifted adults produced significantly more dead eggs, and had slightly less total progeny, than the *Cer1(-)* adults (Figure 7I, 7J). Thus, under the moderate stress of 25°C, wild-type animals pre-loaded with capsids appear to be less fertile than *Cer1(-)* adults.

### *Cer1* capsids traffic toward nuclei in association with dynamic or labile microtubules

**Figure 8:**
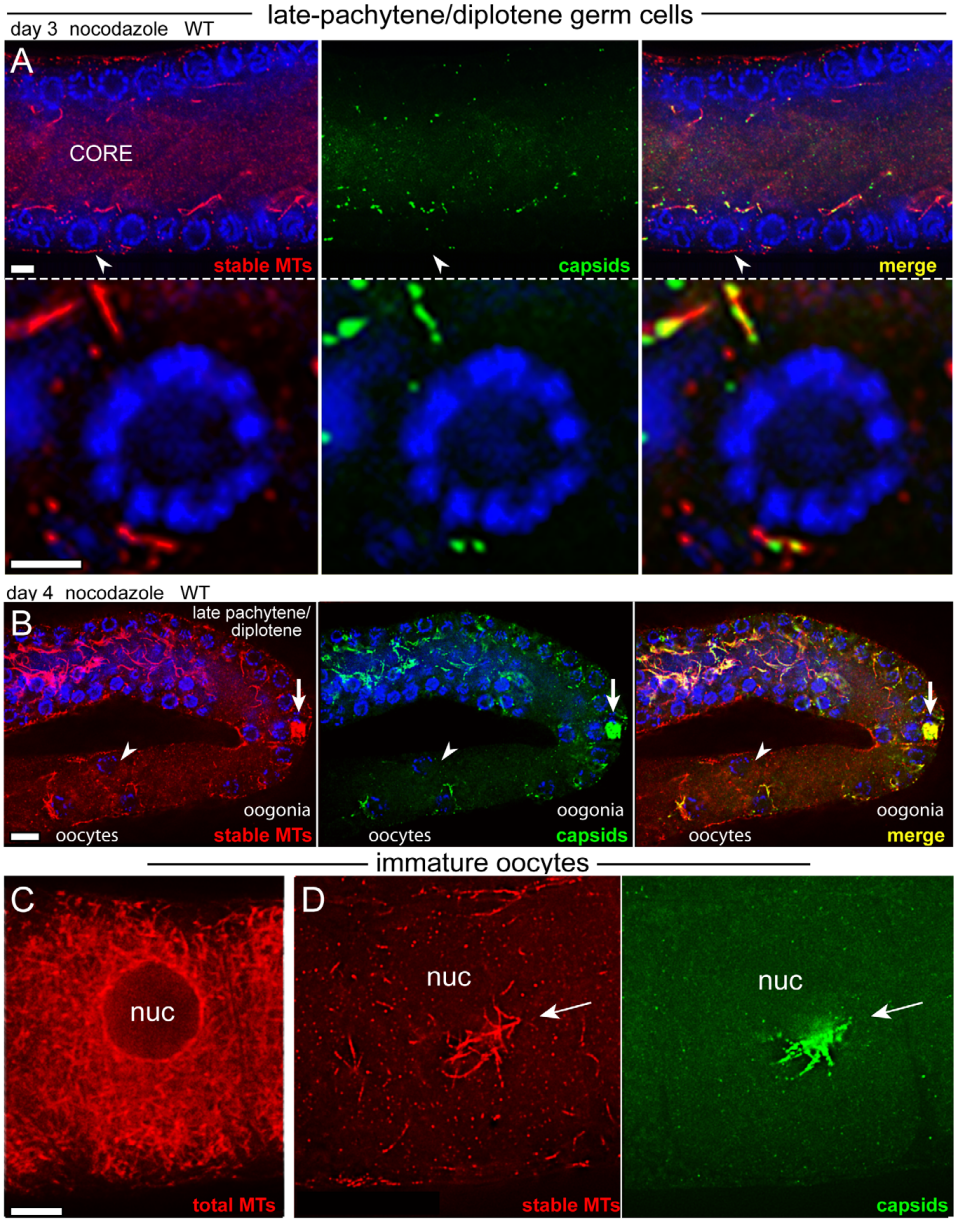
Capsid localization near late-pachytene nuclei requires inhibitor-sensitive microtubules. (A) Late-pachytene/diplotene region of a nocodazole-treated, day 3 wild-type gonad showing the near absence of stable microtubules and capsids; the arrowhead indicates a germ cell shown a higher magnification in the lower panels. Compare these images with the abundant microtubules and capsids in the same region of untreated gonads (Figure 6A, boxed region, and Figure 5G, respectively). (B) Low magnification image of a nocodazole-treated, day 4 wild-type gonad, oriented as in Figure 6D. Note the rare example of a large aggregate of stable microtubules and capsids in an early oogonium. Because oogonia in this region begin to receive cytoplasmic flow from the gonad core, it is possible that this focus broke off from the mid-pachytene region, rather than surviving the normal developmental progression through late pachytene. Note also that many of the oogonia and early oocytes lack crescents of capsids (arrowhead; compare with Figure 3A), and the few crescents of capsids co-localize with stable microtubules. (C) Untreated, wild-type oocyte showing enrichment of microtubules near the nuclear envelope. (D) Nocodazole-treated, wild-type oocyte showing a focus of stable microtubules and a crescent of capsids. Oocytes shown in both panels occupied the -5 position in the ovulation sequence. Scale bars: A (5 µm), B (10 µm), C (8 µm).

Stable microtubules are present in the gonad core throughout the early to mid-pachytene region, but disappear abruptly as germ cells exit pachytene (Figure 6D, 6G, and Figure 8A). Similarly, the giant aggregates of stable microtubules in older adults nearly always disappear by late pachytene/diplotene: From over 160 nocodazole-treated gonads examined, only one contained a giant aggregate of stable microtubules and capsids in this region (arrow in Figure 8B). Thus, stable microtubules appear to break down, or become destabilized, at the same stage that capsids move out of the core and into germ cells (Figure 5E, and Supplemental Video S1). We found that nocodazole treatment sharply reduced the number of capsids in late- pachytene/diplotene germ cells (compare Figure 8A with Figure 5G). Thus, the movement of capsids into germ cells is dependent on microtubules, but these microtubules are not resistant to nocodazole.

The stable microtubules disappear in late pachytene/diplotene germ cells, but reappear in late oogonia and immature oocytes where they are concentrated in a focus near the nuclear envelope (arrow in Figure 6H and in Figure 8D; compare with the untreated oocyte in Figure 8C). This focus of stable microtubules colocalizes with the nuclear-associated crescent of capsids (Figure 8D, see also Figure 4F). Similar crescents of capsids exist in untreated, earlier oogonia that contain few or no stable microtubules (Figure 3A, and Figure 6D), and these latter crescents are disrupted by microtubule inhibitors (arrowhead in Figure 8B). Together, these results suggest that (1) a focus of labile microtubules develops on or near the nuclear envelope after germ cells exit pachytene, (2) that microtubules in the focus gradually stabilize as oogonia develop into immature oocytes, and (3) that capsids accumulate at the focus before and after microtubule stabilization. Finally, the stable microtubules disappear and the capsids disperse in mature oocytes (Figure 6D, 6H). Most capsids eventually disappear in mature oocytes, but at fertilization the remaining capsids aggregate at the meiosis I spindle as described above. We conclude that capsids are associated with microtubules throughout the gonad and in fertilized eggs, and that the various patterns of capsid localization reflect changes in microtubule stability and patterning.

## Discussion

### Temperature-dependence of *Cer1* expression

Nematode germ cells, like those in other animals, presumably have multiple layers of defense against transposons. For example, studies primarily from *Drosophila* and mice have provided an elegant model for how germ cells recognize and silence transposons: If replicating transposons integrate into chromosomal traps called piRNA clusters, it primes the synthesis of small, anti-sense RNAs (piRNAs) that can target both the original transposon and closely-related transposons [44], [45]. Thus, novel transposons entering a naïve host have limited windows of opportunity to replicate before the silencing machinery identifies them. In exploit this window, nematode retrotransposons likely require strategies to traffic through, or bypass, both nuclear pores and P granules.

VLPs have not been reported previously in *C. elegans* germ cells, and only a few were observed in our initial electron microscopic survey of several wild strains. However, we showed that the expression of *Cer1*, a Gypsy/Ty3 retrotransposon, is temperature sensitive, such that very few VLPs are present at typical culture temperatures of 20°C to 23°C. Because previous analyses of spontaneous mutations in *C. elegans* were done at 20°C, it will be important in future studies to re-evaluate whether *Cer1* is capable of transposition in the N2 strain using mated, older adults cultured at 15°C. Temperature-dependent expression appears to be a widespread feature of *Cer1* retrotransposons, as a similar dependence was seen in multiple wild strains with different *Cer1* insertion sites. There are several possible rationales why *Cer1* expression is elevated at low temperatures, the simplest being that 15°C best approximates the average temperature of *C. elegans* in its natural environment. For example, average annual soil temperatures recorded 10 cm below the surface at sites in Florida, Montana, Oregon, Michigan, Tennessee, Arizona, Alaska were 22.5, 12.1, 12.8, 8.2, 15.7, 19.5, and 0.8°C, respectively [46]. In addition, our temperature-shift experiments on older N2 adults suggest that expression at 25°C might exacerbate phenotypes associated with capsid accumulation.

*Cer1* capsids cannot form at 25°C because GAG and POL proteins are not expressed at that temperature. *Cer1* produces an apparently unspliced 8 kb mRNA that can encode GAG and POL proteins as a single polypeptide, and a spliced 2.4 kb mRNA that encodes a novel protein. The 2.4 kb mRNA is abundant at both 15°C and 25°C, however, the 8 kb mRNA only accumulates in the cytoplasm at 15°C. It does not appear that small RNA pathways prevent the cytoplasmic accumulation the 8 kb mRNA, as all of the mutants tested to date lack capsids at 25°C, similar to wild-type animals.

Splicing is essential for most cellular mRNAs to be exported from the nucleus. However, retrotransposon and retroviral replication requires export of a non-spliced, genomic RNA. Retroviruses can achieve this by structural motifs in the genomic RNA that bind host export factors directly, or by first producing proteins from spliced mRNAs that facilitate the subsequent export of non-spliced, genomic RNA. For example, the HIV Rev protein is the product of a spliced mRNA, and binds to the host export factor CRM-1 as well as to a structured motif (the Rev-response element) in the HIV genomic RNA [47], [48]. The 8 kb *Cer1* mRNA is about the same size as the predicted *Cer1* genomic RNA, and thus might serve a dual function as genomic RNA, or at least face similar challenges in nuclear export. Because the novel protein encoded by *Cer1* shares some peptide sequences with GAG, and one of the expected functions of GAG is to bind and package the genomic RNA, we speculate that this novel protein might similarly bind the 8 kb RNA and regulate its splicing or export.

### *Cer1* capsids appear to target meiotic germ cells

The localization pattern of *Cer1* capsids described here for the N2 strain appears very similar to that in other wild strains that express *Cer1*. Thus, this pattern is likely to be relevant for considering how *Cer1* accesses germ cell chromatin. *Cer1* does not appear to bypass P granules and the nuclear envelope by targeting dividing cells in larvae or adults: *Cer1* is not expressed in L4 or earlier larvae, where all germ cells divide mitotically, and *Cer1* is not expressed in the mitotic niche of adult hermaphrodite or male gonads. We found that *Cer1* and *Cer1*-related retrotransposons conserve at least one predicted E2F binding site in their LTRs, and that this site appears important for germline expression. E2F activator proteins have well-studied roles in promoting the mitotic cell cycle in mammals and *Drosophila*, and DNA tumor viruses de-repress E2F protein complexes to drive S-phase DNA synthesis [49]. However, *C. elegans* EFL-1/E2F does not appear to function in mitosis, and instead regulates germ cell expression of numerous genes that control oogenesis and early embryogenesis [34]. Indeed, the present network of EFL-1/E2F-regulated genes in *C. elegans* germ cells might have expanded by the past transposition of *Cer1* family members, much as endogenous retroviruses appear to have seeded p53 binding sites in the human genome [50].

If *Cer1* has not evolved to target mitotic germ cells in adult or larval gonads, it might instead target the mitotic germ cell precursors in early embryos; these precursors have rapid cycle times of only 15–25 minutes, compared to 16–24 hours for adult germ cells [51], [52]. For this strategy, however, it would seem counterproductive for *Cer1* capsids to bind microtubules. If capsids simply did not bind microtubules, they would be transported passively into oocytes by actin-dependent cytoplasmic streaming, and at fertilization would be further transported directly into the embryonic germ cell precursors by a second wave of actin-dependent cytoplasmic flow [27], [53]. Instead, the anchoring of *Cer1* capsids to stable microtubules in the gonad core prevents many, if not most, capsids from ever reaching embryos produced by self-fertilization: Each arm of the hermaphrodite gonad can produce about 1000 oocytes, but only produces about 150 sperm. Thus, adults run out of sperm and can no longer self-fertilize at about the same time that peak numbers of capsids accumulate in the gonad core.

Most capsids in both *C. elegans* and *C. japonica* disappear in mature oocytes, suggestive of host defenses that prevent capsids from entering embryonic cells. For example, oocyte cytoplasm might contain specific proteases that have evolved to recognize, and degrade, capsids. As second possibility is that oocyte cytoplasm triggers the disassembly of capsids. The oocyte cytoplasm contains a very high level of FG-repeat nucleoporins such as NPP-9/RanBP2/Nup358 [24], which presumably is necessary to support the assembly of nuclear pores during the rapid proliferation of early embryonic cells. In addition, oocytes contain high levels of FG-repeat P granule proteins that likely contribute to the dynamic growth, shrinkage and fusion behaviors of detached, cytoplasmic P granules [54]. If *Cer1* capsids, like HIV capsids, target FG-repeat proteins on the nuclear pores, large quantities of such proteins in the cytoplasm might effectively become decoys that trigger premature capsid disintegration.

While *Cer1* capsids are not enriched in any mitotic germ cell population, they are expressed abundantly throughout the pachytene stage of meiosis. However, the vast majority of capsids remain in the gonad core until germ cells begin to exit pachytene, when most capsids transfer into germ cells and near nuclei. As germ cells exit pachytene, they begin to increase in size and eventually become large oocytes. There is a concomitant increase in nuclear size, with the addition of nuclear pores that lack P granules and the gradual removal of existing P granules. Thus, capsids converge on germ nuclei when large numbers of P granule-free nuclear pores first become available. Such a strategy would likely provide only a narrow window of time for nuclear entry and integration, as the post-pachytene germ cells cease transcription and hypercompact their chromatin as they differentiate into oocytes (Figure 1C).

### Navigating the *C. elegans* gonad by changes in microtubule patterning and stability

By electron microscopy, we detected *C. japonica* VLPs in the early- to mid-pachytene region, similar to *Cer1* capsids in *C. elegans* gonads. However, the *C. japonica* VLPs first appear, and then remain, on P granules. As newly exported mRNA traffics directly through P granules [24], this localization might be an efficient strategy for capsid proteins to capture and package viral RNA. In addition, P granule localization maintains capsids by the nuclear envelope, potentially allowing a constant surveillance for free nuclear pores. In contrast, *Cer1* capsids in *C. elegans* appear to traffic toward nuclei from initial positions in the gonad core. Particles the size of the 80 nm *Cer1* capsids are not expected to move appreciably without some type of force-generating machinery; studies in other systems have shown that dextran particles greater than 20 nm are essentially immobile in cytoplasm (see [55] and references therein). Actin-dependent streaming moves cytoplasm through the core of the gonad and into late oogonia, but does not appear to transport cytoplasm into the earlier germ cells that show perinuclear accumulation of capsids [27]. We found that *Cer1* capsids are associated with microtubules in all regions of the gonad, suggesting that changes in the microtubule cytoskeleton underlie changes in capsid localization. For example, *Cer1* capsids are dispersed uniformly throughout the cytoplasm of mature oocytes just prior to fertilization. At fertilization, however, the microtubule cytoskeleton reorganizes to form the meiotic spindle and capsids converge at both poles of the spindle.

Viruses often exploit the microtubule cytoskeleton to locate host nuclei [56], [57]. In many cell types, microtubule minus ends are anchored by the centrosome, which functions as the major microtubule organizing center (MTOC). Because the centrosome is often adjacent to the nucleus, viruses can traffic from the cell periphery toward the nucleus by hijacking minus-end directed microtubule motor proteins; upon release from the microtubule, the virus has a high probability of encountering a nuclear pore. For example, herpes simplex virus, HIV-1, and adenovirus each use the minus-end directed motor protein dynein to navigate host cells [56], [57]. *C. elegans* gonads contain dense populations of microtubules that include subpopulations we term basket microtubules and core microtubules. However, the centrosome appears to lose its MTOC function once germ cells exit mitosis, and the centrosome itself is eliminated during oogenesis [28]. Instead, the germ cell plasma membrane appears to function as an MTOC for basket microtubules [28], and we consider it likely that the core plasma membrane acts as an MTOC for core microtubules. Animal cells typically have dynamic microtubules (t_1/2_ = ∼10 minutes) as well as long-lived, stable microtubules (t_1/2_>1 hour) [58]. In the *C. elegans* gonad, we showed that some basket and core microtubules appear exceptionally resistant to concentrations of microtubule inhibitors that rapidly depolymerize known, dynamic populations of microtubules, such as those in mitotic germ cells and in the most mature oocytes. Because capsids appear to accumulate over time on the stable microtubules, we argue that these same microtubules are long-lived in vivo.

**Figure 9:**
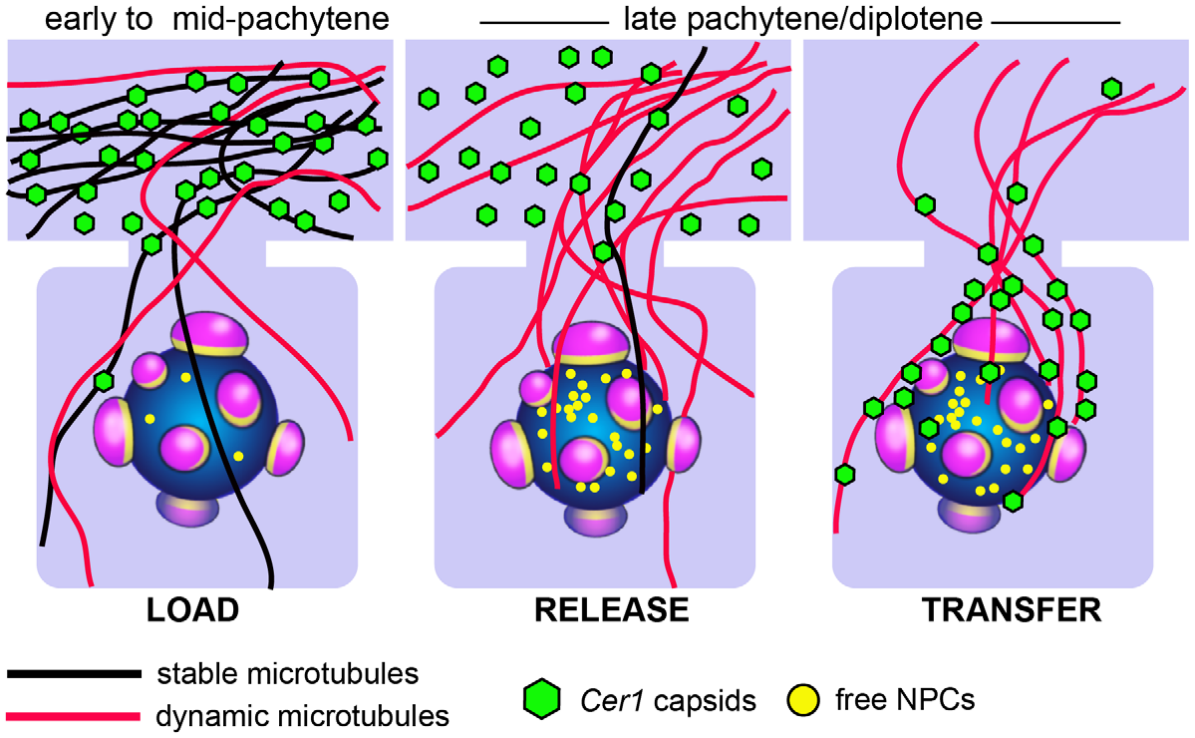
Load-Release-Transfer model for capsid traffic toward late-pachytene/diplotene nuclei. Cartoon of capsids and microtubules as germ cells progress into late pachytene/diplotene. Capsids that had accumulated on stable microtubules (black) in the core (top) are released as these microtubules depolymerize. The released capsids transfer to surrounding labile, or dynamic, microtubules (red) and traffic into germ cells. In an alternative model, capsids could remain on the original microtubule as it becomes destabilized. Traffic could involve microtubule dynamics, or microtubule motor proteins.

We propose a “load-release-transfer” mechanism as a working model for how *Cer1* capsids concentrate near post-pachytene germ nuclei (Figure 9). First, capsids that form in the early- to mid-pachytene region load onto surrounding microtubules in the core. Capsids might initially associate equally with dynamic and stable microtubules, but over time would accumulate on the long-lived, stable microtubules. Early to mid-pachytene germ cells are small with relatively little cytoplasm, but microtubules extending from these cells into the core represent a much larger, cognate surface for capsid accumulation. Cytoplasmic dynein is required for normal levels of capsid accumulation in the core, but we do not yet know whether dynein mediates capsid binding to microtubules, or whether capsids can traffic on the stable microtubules.

The number of capsids loaded onto stable microtubules increases as germ cells progress through pachytene, often resulting in the bundling or aggregation of stable microtubules in older adults. We consider it likely that the stable microtubules provide the architectural framework for the gonad core throughout the pachytene region, and must be destabilized for germ cells to increase in size as they exit pachytene. The abrupt change in microtubule stability releases the accumulated capsids into a relatively small region of core cytoplasm. There, many capsids transfer to dynamic microtubules, and move into germ cells, before being swept proximally by flow. The transferred capsids might traffic toward nuclei in association with microtubule motor proteins, or by microtubule dynamics. Although we have not yet determined the polarity of microtubules in this region, it seems likely that many are nucleated at or near the nuclear envelope and could concentrate capsids: Microtubules in general are enriched by the nuclear envelope of those cells, and a focus of stable microtubules develops close to the envelope where capsids accumulate. Thus, we hypothesize that a new, nuclear-associated MTOC forms as cells exit pachytene, and microtubules associated with this site gradually stabilize as germ cells mature into oogonia. This MTOC is not associated with the centrosome per se, which is eliminated during oogenesis, but might be associated with a remnant of the centrosome or with centrosome-derived proteins.

The present study demonstrates that the genetics and cell biology of *C. elegans*, one of the best understood models for animal development, can be used to study host-pathogen interactions between retroelements and germ cells. Because viruses necessarily rely heavily on host functions, their analysis has provided significant insights into host biology in many systems. Here, our analysis uncovered stage-dependent changes in the microtubule cytoskeleton of *C. elegans* germ cells, posed new questions about how microtubule stability is established and regulated, and showed that capsid localization can be used as a proxy for the distribution of stable microtubules. Further dissection of *Cer1* expression should provide insights into RNA splicing and export in germ cells, EFL-1/E2F-regulated transcription, and inform strategies for building *Cer1*-based vectors for temperature-inducible, germ cell-specific gene expression. Although the various small RNA pathways do not silence *Cer1* expression in the N2 strain at 15°C, we anticipate that analysis of *Cer1*-containing wild strains that lack GAG expression should reveal how this element is targeted for silencing in wild populations.

## Materials and Methods

### RNA analysis

For Northern analysis, synchronous hermaphrodites grown from bleached eggs were grown at 20°C until the L4 stage, then either shifted to 15°C or 25°C for 48 hours. About 1 ml of packed adults from each culture was rinsed in PBS twice, then resuspended and homogenized in Trizol (Invitrogen), followed by treatment with DNAse and RNA precipitation as per the manufacturer's instructions. The RNA was checked for purity by running samples on agarose gels. PolyA-containing RNA was selected using an Oligotex mRNA midi Kit (Qiagen) following the kit protocol. Probes were prepared using Amersham Ready-to-Go DNA labeling beads-dCTP (Amersham), and unincorporated nucleotides were removed with NucAway Spin Columns (Ambion), both following product protocols. RNA blotting and hybridization followed published protocols [59], except with the suggested alternate buffer [Prehybridization/hybridization: 7% SDS, 0.5 M NaHPO45, 1 mM EDTA; wash #1: 5% SDS, 40 mM NaHPO4, 1 mM EDTA; wash #2: 1%SDS, 40 mM NaHPO4, 1 mM EDTA]. Nylon membrane was used for blotting (BioRad) as per the manufacturer's protocol. For RT PCR, cDNAs were generated using SuperScript One-Step RT PCR (Invitrogen) and Platinum Taq (Invitrogen). Products were gel purified using QiaQuick Gel Extraction Kit (Qiagen) and sequenced by the FHCRC Genomics Resource. The following primer sequences were used for the analysis summarized in Figure 2A: SL1(5′-GTTTAATTACCCAAGTTTGAG-3′), KE409F(5′-CGCCTTTGAGTTTGCAATCGTTT-3′), KE402F(5′-AAATCTGAAGAATGGAGGTGAACGAGGGACAGGATA-3′), KE411F (5′-TTGGATAGTGGTGCTAGTATTTC-3′), KE413F(5′-GACTTCGTCATTGACACCTTATC-3′), KE416R(5′-TCCGATTTCACCATTAGCAAAC-3′), KE401BR(5′-ATAACACCTCAAAGCCTCCTG-3′), KE415R(5′-GATAAGGTGTCAATGACGAAGTC-3′), KE402CR(5′-TTCAAAGAAACAGGCAGAGCG-3′).

The in situ procedure was modified from the protocol of G. Broitman-Maduro and M. Maduro (http://www.faculty.ucr.edu/~mmaduro/) as previously described [24]. DIG-labeled ssRNA probes were made by in vitro transcription using DIG RNA Labeling Kit (Roche), using as template a PCR product amplified from genomic DNA with the following primer sequences: *rt* probe (5′-AGGTGATTGGGAAAGGAGAG-3′, 5′-GCGATTGTAGCGTTGCTTCA-3′), *gag* probe (5′-TCCCAGGTCCAGACGAATAG-3′, 5′-GCTCTCTCGAGATATTCTGAG-3′)

### RNAi experiments

Unless noted otherwise, RNAi experiments used worms cultured at 20–23°C and dsRNA feeding strains from the Ahringer library as described [60]. Third larval stage (L3) *alg-2(ok304)* animals were fed on *alg-1*(F48F7.1 dsRNA) until adulthood. For analysis of *Cer* elements, N2 worms were fed on dsRNA-expressing bacteria for 3 generations before analysis of adults using the following library strains: *Cer1* (F44E2.2 dsRNA), *Cer4* (T23E7.1 dsRNA), and *Cer5* (T03F1.4 dsRNA). Sequences from the remaining *Cer* elements were PCR amplified from genomic DNA using the following primer pairs then cloned into feeding vector pPD129.36 as described [61]: *Cer2* (5′-GGACGAGCTTATCGTGTTCC-3′, 5′-CAACTCAACGAGCCATCTCC-3′), *Cer3* (5′-ATGAGAGAGCTCAGTTACAAGC-3′, 5′-GGTTCTACTAGTATAAGCTATCG-3′), *Cer6* (5′-ATGACGGTTAAGGGTCGAGTT-3′, 5′-CGATGAATCCCAGAAACGAGAT-3′), *Cer13* (5′-TCTTTCAAGGCATCTCAGCG-3′, 5′-AACCTCGGTCCAATTGATCC-3′ plus 5′-TTCTCAACAGCGTCTCCATCG-3′, 5′-CAGCTGTCCGATCTGTTTCAC-3′.

### Immunocytochemistry and microscopy

The following antibodies were used: αTubulin (1∶1 YL1/2 and YOL3/4; Abcam), αPGL-1 [62], αHIM-4 [63]. αGAG was generated in the FHCRC Hybridoma Production Facility using bacterially expressed, GST-tagged GAG following published protocols [64]. GAG protein-coding sequences from nucleotide positions 1789–2748 were fused in frame at the C-terminus of GST using pGEX-6P-1 (Amersham), and purified from Rosetta (DE3) pLyS cells (Novagen). Approximately 500 hybridoma colonies were identified that screened positively against GST-GAG by the ELISA screening test. Cell line P3E9 (αGAG) was obtained by re-screening all of the positive hybridoma cell supernatants by indirect immunofluorescence microscopy of fixed wild-type and *Cer1(RNAi)* gonads.

### Inhibitor experiments

Worms were collected in drops of M9 buffer [65] on a taped microscope slide and rinsed in three or more changes of M9 to clear bacteria, then rinsed in two changes of gonad culture media (GCM: 50% Leibovitz L-15 medium (GIBCO), 10% Fetal Calf Serum (GIBCO), 5.6% sucrose, 2 mM MgCL_2_). Slides containing 20–30 worms in a drop of GCM were placed on a precooled dissecting microscope stage at 8–10°C; cooling was used to slow animal movements to facilitate dissection. Worms were cut immediately with a scalpel blade, just behind the pharynx to release the gonad, and the slides were removed to room temperature; the elapsed time on the cooled stage did not exceed 2 minutes. An equal volume of 15°C GCM was added that contained 2× nocodazole (Sigma) plus 1 mM levamisole (Sigma) in GCM. Nocodazole was diluted fresh each time from a 5 mg/ml stock in dimethylsulfoxide kept at −20°C. The levamisole was added after dissection to prevent damage to the gonad from the contraction of attached muscles during incubation with the inhibitor. The slide with gonads plus inhibitor was placed on a pre-cooled block at 15°C, and transferred to a 15°C incubator for the designated time.

### Fixation, immunostaining, and microscopy

MeOH fixation was used for images shown in Figure 3 and for experiments listed in Tables 3 and 4 as follows. Worms were dissected on microscope slides in a small drop of M9 buffer and covered with a coverslip. The slide was frozen on dry ice, the coverslips removed, and the slide immersed in −20°C MeOH (5 minutes) before rinsing in three changes of PBS for 5 minutes each. Slides were incubated with antibody overnight at 4°C. A two step formaldehyde fixation [66] was modified for experiments to simultaneously localize *Cer1* GAG and microtubules as follows. Worms were dissected as for the nocodazole experiments above, except that gonad buffer (GB: 75 mM HEPES (6.9), 1.2% sucrose, 40 mM NaCl, 5 mM KCL, 2 mM MgCL2, 1 mM EGTA) was substituted for GCM. After dissection, an equal volume of fixation buffer #1 [6% formaldehyde, 75 mM HEPES (6.9), 40 mM NaCl, 5 mM KCL, 2 mM MgCL2, 1 mM EGTA] was added for 2 minutes, then two equal volumes of fixation buffer #2 [3% formaldehyde, 30 mM Sodium Borate (11), 2 mM MgCl2] were added briefly before removing the combined fixative and adding fixation buffer #2 for 7 minutes. The gonads were rinsed briefly with PBS, permeabilized in PBS plus 0.3% Triton X-100 (Sigma) for 10 minutes, then rinsed several times in PBS and immunostained overnight at 4°C.

Widefield images (Figure 2B–2D, Figure 3A–3E) were acquired with a Nikon Eclipse 90i upright microscope using 40× (Plan Fluor, 1.4 NA) and 60× (Plan Apo VC, 1.4 NA) lenses and a Retiga-4000DC camera (QImaging). Images were exported to Adobe Photoshop for cropping, reorientation, and contrast and brightness adjustments. All other images were acquired with a DeltaVision microscope and processed using deconvolution software (Applied Precision). All images shown are single plane, except for Figure 5A and Figure 5E, which are two plane (0.2 µm) projections to visualize ring channels.

For electron microscopy, L4 hermaphrodites grown at 20°C–23°C were selected and allowed to develop to young adults for 24–48 hours. For male/female strains, L4 females were collected and mated with males. Gonads were dissected from 40–60 adults, then fixed with glutaraldehdye and osmium tetroxide and processed for electron microscopy as described [20]. Grids were viewed with a JEOL JEM-1230 transmission electron microscope. To avoid scoring individual germ cells and gonads more than once in the initial survey, each gonad was numbered and scored in a single, representative thin section through the middle of the core. Germ cells typically were examined at 4,000× to 6,000×, and candidate VLPs were photographed at 8,000× with a Gatan UltraScan 1000 CCD camera. For each sample, germ cells were examined in the distal, mitotic region, and at all stages of meiosis through oogenesis. The gonad core contains large numbers of vesicles of various shapes that might be confused with VLPs, however the cytoplasm around germ nuclei is relatively devoid of organelles. Thus, quantitation was performed only on germ “cells” up to the ring channel, although the core was inspected in many gonads.

## Supporting Information

Figure S1 ***C. japonica*** **VLPs are associated with P granules in adult gonads, and microtubules in somatic cells.** Electron micrographs of VLPs in *C. japonica* (A–F) and *C. elegans* (G–H). *C. japonica* images show VLPs (white arrows) clustered on P granules in an adult female (A), and in adult males (B and C); small black arrows indicate nuclear pores. (D–F) *C. japonica* embryos showing VLPs in differentiating somatic tissues: hypodermis (hyp, panel D), muscle (panel E), and intestine (int, panel F). Insets show higher magnification examples of embryonic VLPs; note association with microtubules in panels D and E (arrows). (G) Examples of *C. elegans* VLPs when they are first detected in the early- to mid-pachytene core of the gonad. Capsids typically appear in small, non-contiguous groups associated with rough endoplasmic reticulum (arrows). (H) Examples of capsids within P granules in late pachytene germ cells; arrows indicate nuclear pores. Scale bars = 0.2 µm.
(TIF)

Figure S2 ***Cer1*** **-family LTRs contain predicted binding sites for EFL-1/E2F.** Alignment of 5′ LTR and PBS sequences from (1) the N2 copy of *Cer1*, (2) a member of a now extinct, *Cer1*-like family of retrotransposons in N2 (cosmid C03F11), and (3) a *Cer1*-like retrotransposon in the *C. japonica* genome (Contig10639; *C. japonica*-7.0.1, http://genome.wustl.edu). Shaded nucleotides are present in two or more LTRs. Boxed regions indicate predicted E2F/EFL-1 binding sites (G/C)GCG(G/C)GAA [34], polyA-addition sites, and a possible Primer Binding Site (PBS) 3′ to the LTR. The sequence TGG (underlined) at the start of the PBS is not complementary to the 3′ end of the *C. elegans* tRNA-Pro gene, but the complementary triplet CCA is added posttranscriptionally to the 3′ terminus of eukaryotic tRNAs. Fragments from additional members of the extinct family of *Cer1-like* retrotransposons are found in the following N2 clones: C18A11, C18H2, C24H12, F07G6, F11A5, F16B12, F28B4, F28H6, F40E3, F54G2, T05E8, T22F7, Y20C6A, Y39B6A, Y59E1A, Y67D8C, Y69A2AR, Y71G12B, Y751H4A, Y82E9BL, ZC513, and ZC53 (http://www.wormbase.org, release WS227, 2011). For example, a family member inserted in the N2 gene F54G2.1 contains a partial 5′ LTR, the PBS, and sequences that can encode a *Cer1*-like GAG and protease.
(TIF)

Figure S3 **Generation of the 8 kb** ***Cer1*** **mRNA requires skipping** ***C. elegans*** **consensus splice sites.** The central black bar represents the *Cer1* sequence (diagrammed at top). Vertical lines above and below the bar indicate candidate 5′ or 3′ intron splice sites, respectively; the height of each line indicates the *C. elegans* splice site prediction score (scale 0.5 to 1.0) using NetGene2 [http://www.cbs.dtu.dk/services/NetGene2] [79], [80]. Vertical lines colored red are the sites used to generate the spliced, 2.4 kb *Cer1* mRNA at 15°C and 25°C (this study). The red boxes represent exon sequences in the 2.4 kb mRNA, and the sites of SL1 trans-splicing and poly(A) addition are indicated by arrows. Note that most of splice sites utilized conform well with *C. elegans* consensus sequences, and must be skipped to generate the 8 kb *Cer1* mRNA. The intron splice sites that generate the fourth exon (3′ site = attcag and 5′ site = gtgag) are less common and fall below the 0.5 score illustrated, but are present in other spliced introns in *C. elegans* [38].
(TIF)

Figure S4 **Aggregates of capsids and microtubules are present in day 3 and older, wild-type adults.** (A–B′). Electron micrographs showing aggregates of capsids and microtubules that resemble the wavy lines of capsids (A) and tangles of wavy lines (B) observed by immunofluorescence; compare with Figure 5C and 7C, respectively. Capsids and microtubules visible in these and/or the adjacent thin sections (not shown) are colored in panels A′ and B′. The inset in panel B shows capsids and microtubules (arrowheads) in the boxed region at higher magnification. Scale bar = 0.15 µm (A, B).
(TIF)

Video S1 ***Cer1*** **capsids relocate near nuclei in post pachytene germ cells.** Movie showing the late-pachytene/diplotene region of a wild-type gonad. The gonad is immunostained for *Cer1* capsids (green), and for the apical membrane (red, HIM-4 staining). The region shown is similar to Figures 5E and 5H. Note that capsids outline the germ nuclei at the periphery of the gonad, but are nearly absent in the gonad core. This movie was built from an 8 micron, Z-stack of deconvolved optical sections (Deltavision) collected near the center of the gonad core. The image stack was rotated using Volocity imaging software.
(MOV)
