# Supplementary figures and images for: C. elegans Germ Cells Show Temperature and Age-Dependent Expression of Cer1, a Gypsy/Ty3-Related Retrotransposon
Source: PLoS Pathog. 2012 Mar 29;8(3):e1002591. doi: 10.1371/journal.ppat.1002591 (PMC3315495; doi:10.1371/journal.ppat.1002591)

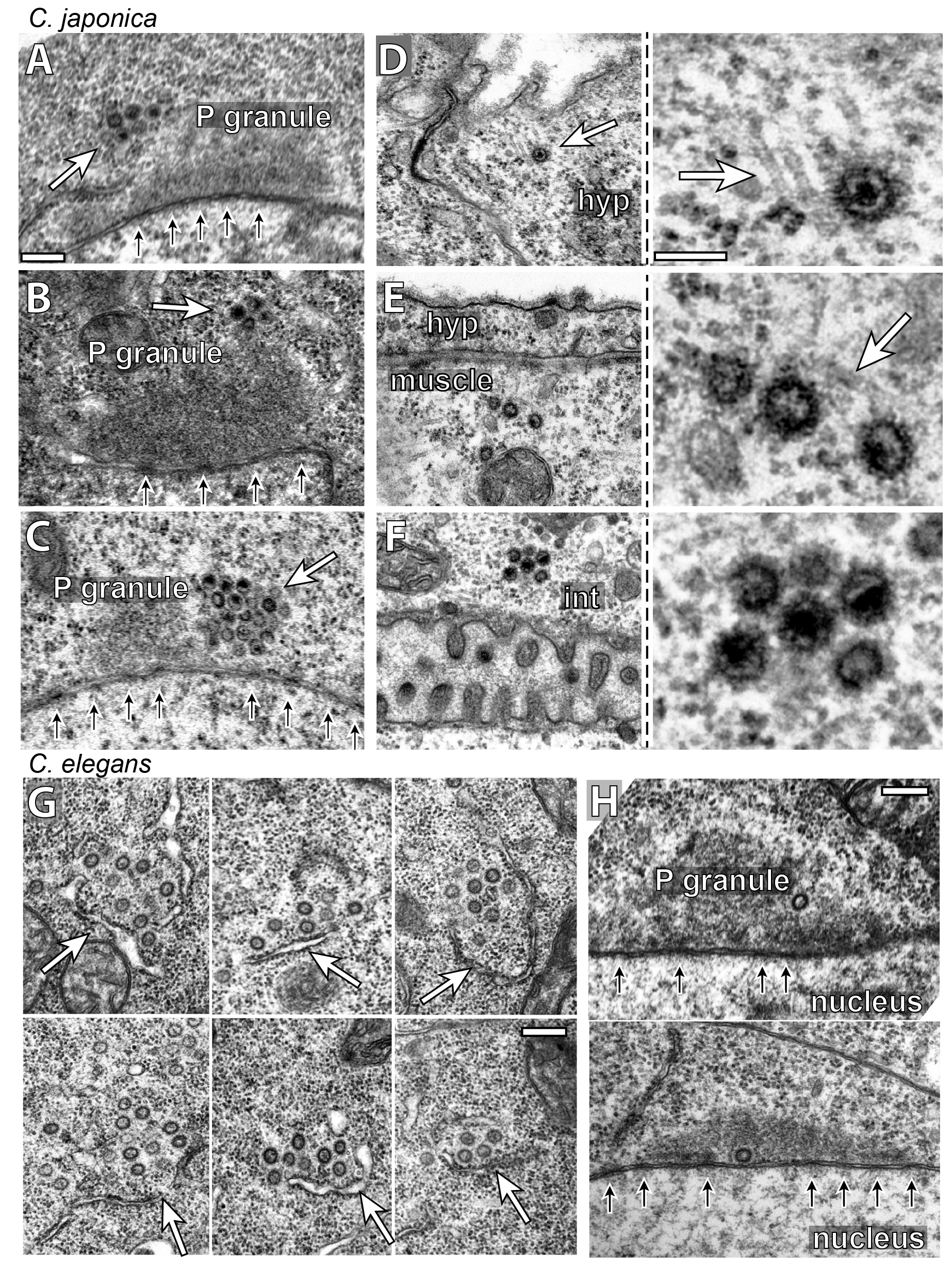

Supplement: Figure S1 — C. japonica VLPs are associated with P granules in adult gonads, and microtubules in somatic cells. Electron micrographs of VLPs in C. japonica (A–F) and C. elegans (G–H). C. japonica images show VLPs (white arrows) clustered on P granules in an adult female (A), and in adult males (B and C); small black arrows indicate nuclear pores. (D–F) C. japonica embryos showing VLPs in differentiating somatic tissues: hypodermis (hyp, panel D), muscle (panel E), and intestine (int, panel F). Insets show higher magnification examples of embryonic VLPs; note association with microtubules in panels D and E (arrows). (G) Examples of C. elegans VLPs when they are first detected in the early- to mid-pachytene core of the gonad. Capsids typically appear in small, non-contiguous groups associated with rough endoplasmic reticulum (arrows). (H) Examples of capsids within P granules in late pachytene germ cells; arrows indicate nuclear pores. Scale bars = 0.2 µm. (TIF) [file ppat.1002591.s001.tif]

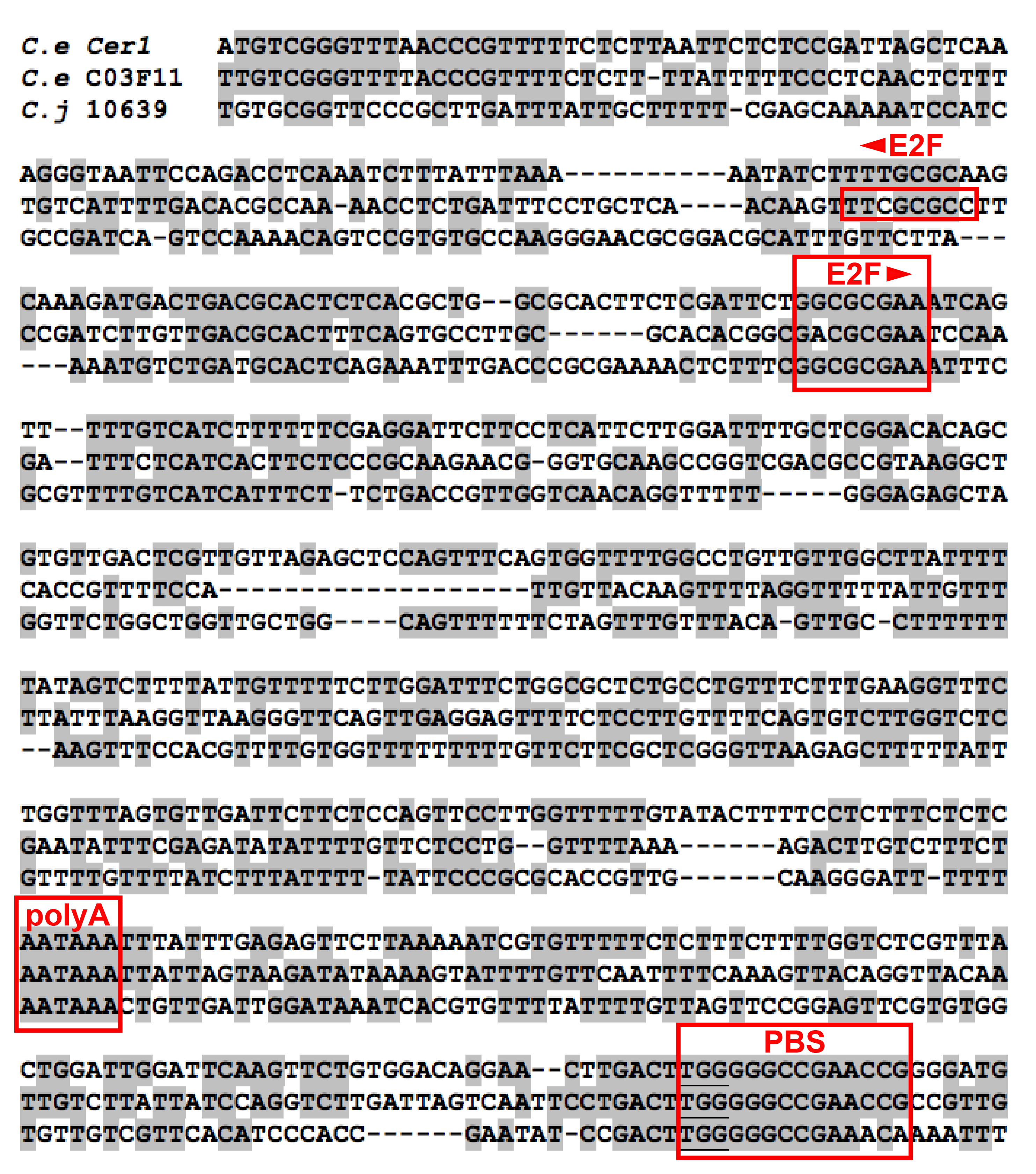

Supplement: Figure S2 — Cer1 -family LTRs contain predicted binding sites for EFL-1/E2F. Alignment of 5′ LTR and PBS sequences from (1) the N2 copy of Cer1, (2) a member of a now extinct, Cer1-like family of retrotransposons in N2 (cosmid C03F11), and (3) a Cer1-like retrotransposon in the C. japonica genome (Contig10639; C. japonica-7.0.1, http://genome.wustl.edu). Shaded nucleotides are present in two or more LTRs. Boxed regions indicate predicted E2F/EFL-1 binding sites (G/C)GCG(G/C)GAA [34], polyA-addition sites, and a possible Primer Binding Site (PBS) 3′ to the LTR. The sequence TGG (underlined) at the start of the PBS is not complementary to the 3′ end of the C. elegans tRNA-Pro gene, but the complementary triplet CCA is added posttranscriptionally to the 3′ terminus of eukaryotic tRNAs. Fragments from additional members of the extinct family of Cer1-like retrotransposons are found in the following N2 clones: C18A11, C18H2, C24H12, F07G6, F11A5, F16B12, F28B4, F28H6, F40E3, F54G2, T05E8, T22F7, Y20C6A, Y39B6A, Y59E1A, Y67D8C, Y69A2AR, Y71G12B, Y751H4A, Y82E9BL, ZC513, and ZC53 (http://www.wormbase.org, release WS227, 2011). For example, a family member inserted in the N2 gene F54G2.1 contains a partial 5′ LTR, the PBS, and sequences that can encode a Cer1-like GAG and protease. (TIF) [file ppat.1002591.s002.tif]

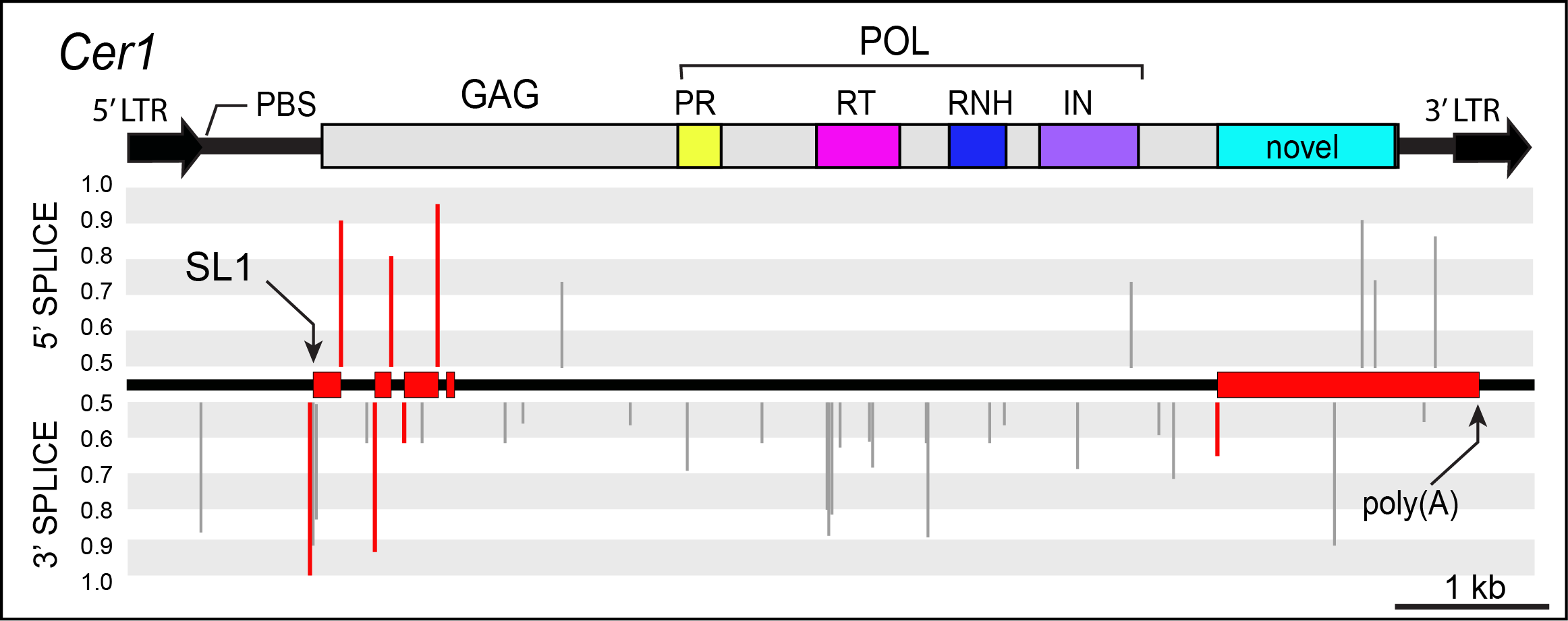

Supplement: Figure S3 — Generation of the 8 kb Cer1 mRNA requires skipping C. elegans consensus splice sites. The central black bar represents the Cer1 sequence (diagrammed at top). Vertical lines above and below the bar indicate candidate 5′ or 3′ intron splice sites, respectively; the height of each line indicates the C. elegans splice site prediction score (scale 0.5 to 1.0) using NetGene2 [http://www.cbs.dtu.dk/services/NetGene2] [79], [80]. Vertical lines colored red are the sites used to generate the spliced, 2.4 kb Cer1 mRNA at 15°C and 25°C (this study). The red boxes represent exon sequences in the 2.4 kb mRNA, and the sites of SL1 trans-splicing and poly(A) addition are indicated by arrows. Note that most of splice sites utilized conform well with C. elegans consensus sequences, and must be skipped to generate the 8 kb Cer1 mRNA. The intron splice sites that generate the fourth exon (3′ site = attcag and 5′ site = gtgag) are less common and fall below the 0.5 score illustrated, but are present in other spliced introns in C. elegans [38]. (TIF) [file ppat.1002591.s003.tif]

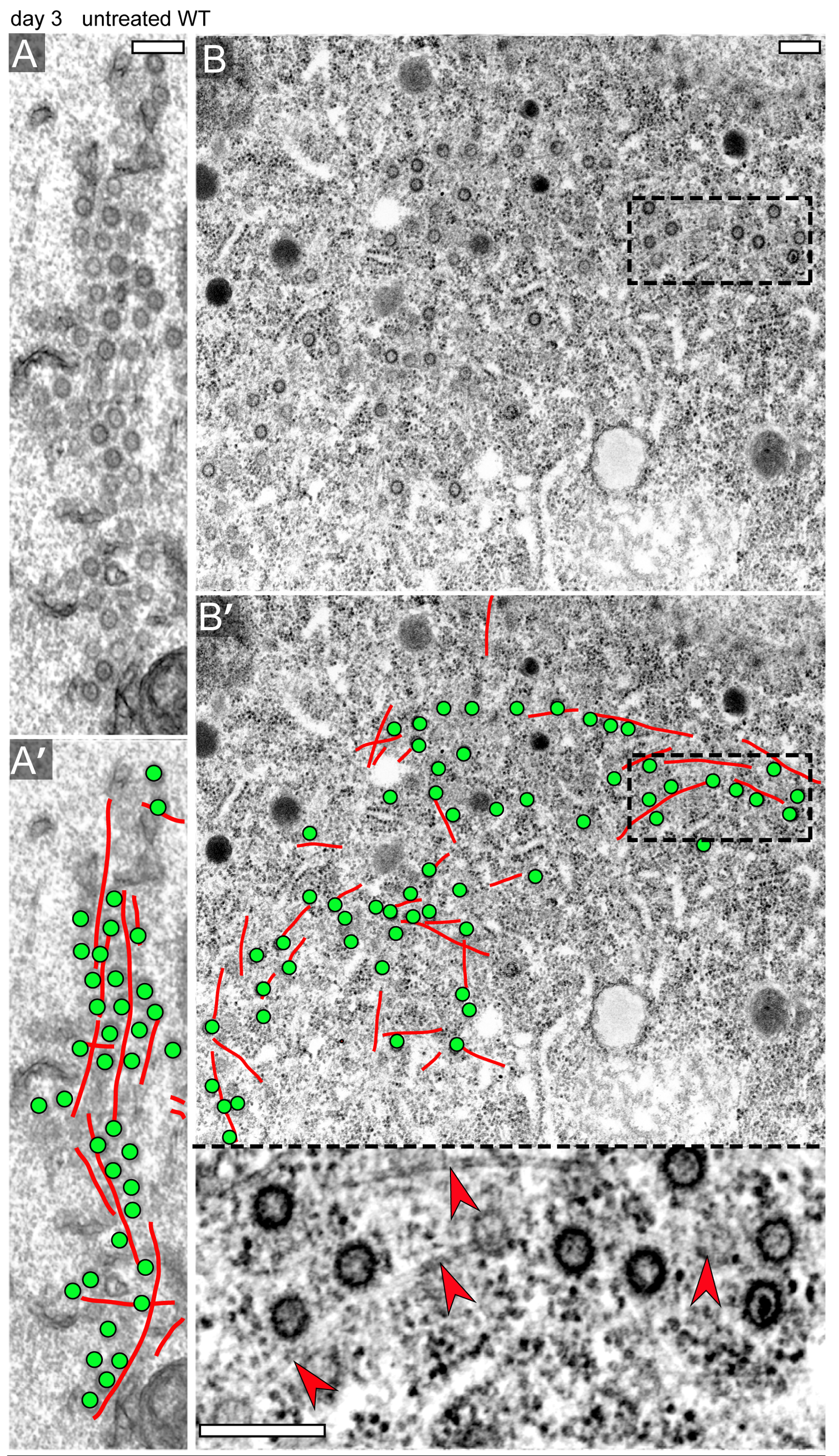

Supplement: Figure S4 — Aggregates of capsids and microtubules are present in day 3 and older, wild-type adults. (A–B′). Electron micrographs showing aggregates of capsids and microtubules that resemble the wavy lines of capsids (A) and tangles of wavy lines (B) observed by immunofluorescence; compare with Figure 5C and 7C, respectively. Capsids and microtubules visible in these and/or the adjacent thin sections (not shown) are colored in panels A′ and B′. The inset in panel B shows capsids and microtubules (arrowheads) in the boxed region at higher magnification. Scale bar = 0.15 µm (A, B). (TIF) [file ppat.1002591.s004.tif]
